# Photosynthesis in a Changing Global Climate: Scaling Up and Scaling Down in Crops

**DOI:** 10.3389/fpls.2020.00882

**Published:** 2020-07-06

**Authors:** Marouane Baslam, Toshiaki Mitsui, Michael Hodges, Eckart Priesack, Matthew T. Herritt, Iker Aranjuelo, Álvaro Sanz-Sáez

**Affiliations:** ^1^Laboratory of Biochemistry, Faculty of Agriculture, Niigata University, Niigata, Japan; ^2^Graduate School of Science and Technology, Niigata University, Niigata, Japan; ^3^Institute of Plant Sciences Paris-Saclay (IPS2), CNRS, INRAE, Université Paris-Saclay, Université Evry, Université Paris Diderot, Paris, France; ^4^Institute of Biochemical Plant Pathology, Helmholtz Zentrum München, German Research Center for Environmental Health, Neuherberg, Germany; ^5^USDA-ARS Plant Physiology and Genetics Research, US Arid-Land Agricultural Research Center, Maricopa, AZ, United States; ^6^Agrobiotechnology Institute (IdAB-CSIC), Consejo Superior de Investigaciones Científicas-Gobierno de Navarra, Mutilva, Spain; ^7^Department of Crop, Soil, and Environmental Sciences, Auburn University, Auburn, AL, United States

**Keywords:** photosynthesis, climate change, crop improvement, -omics, phenotyping, modeling

## Abstract

Photosynthesis is the major process leading to primary production in the Biosphere. There is a total of 7000bn tons of CO_2_ in the atmosphere and photosynthesis fixes more than 100bn tons annually. The CO_2_ assimilated by the photosynthetic apparatus is the basis of crop production and, therefore, of animal and human food. This has led to a renewed interest in photosynthesis as a target to increase plant production and there is now increasing evidence showing that the strategy of improving photosynthetic traits can increase plant yield. However, photosynthesis and the photosynthetic apparatus are both conditioned by environmental variables such as water availability, temperature, [CO_2_], salinity, and ozone. The “omics” revolution has allowed a better understanding of the genetic mechanisms regulating stress responses including the identification of genes and proteins involved in the regulation, acclimation, and adaptation of processes that impact photosynthesis. The development of novel non-destructive high-throughput phenotyping techniques has been important to monitor crop photosynthetic responses to changing environmental conditions. This wealth of data is being incorporated into new modeling algorithms to predict plant growth and development under specific environmental constraints. This review gives a multi-perspective description of the impact of changing environmental conditions on photosynthetic performance and consequently plant growth by briefly highlighting how major technological advances including omics, high-throughput photosynthetic measurements, metabolic engineering, and whole plant photosynthetic modeling have helped to improve our understanding of how the photosynthetic machinery can be modified by different abiotic stresses and thus impact crop production.

## Introduction

Owing to the expected increase in the world’s population, yields of major crops must increase by over 70% in the next 30 years to sustain human requirements (FAO, 2009) and this should be attained without increasing the use of arable land and detrimental effects on nutritional quality while limiting the use of fertilizers and pesticides This means that breeders must increase crop yield at a rate of +2.4% per year, while the current rate is only +1.3% (FAO, 2009). In addition, abiotic stresses such as heat, drought, and flooding among others will tend to decrease yields up to 50% by 2050, if management techniques such as precision irrigation and breeding for abiotic stress tolerance are not implemented (Bierbaum et al., 2007).

Photosynthesis is a complex process that for simplification can be divided into light reactions driven by electrons passing through different protein complexes associated with chloroplast thylakoid membranes and the Calvin cycle reactions of photosynthetic CO_2_ fixation taking place in the chloroplast stroma (Renger, 2007). In the light, the photosynthetic electron transfer chain consisting of photosystem II (PSII), the cytochrome *b_6_f* complex (cytb_6_f), photosystem I (PSI), and the free electron carriers plastoquinone (PQ) and plastocyanin, lead to the production of ATP and NADPH that fuel the Calvin-Benson cycle (CBC) and other assimilatory processes (Rochaix, 2011; Foyer et al., 2012). Three main stages operate during the CBC reactions namely carbon fixation, reduction, and regeneration. In all plants, CO_2_ can be fixed by ribulose-1,5-bisphosphate carboxylase/oxygenase (RuBisCO), an enzyme catalyzing the carboxylation of ribulose-1,5-bisphosphate (RuBP) and leading to two molecules of 3-phosphoglycerate (3-PGA). Instead of CO_2_, RuBisCO can also add O_2_ to RuBP, resulting in one molecule each of 3-PGA and 2-phosphoglycolate (2-PG). Since 2-PG is toxic, it has to be removed in a metabolic pathway called photorespiration that is not only energy demanding, but also leads to a loss of carbon in the form of CO_2_. Thus the efficiency of photosynthesis can be substantially decreased under environmental conditions favoring photorespiration (Ehleringer et al., 1991) and this would be associated with factors altering CO_2_ entry and diffusion within the leaf such as stomatal density and aperture.

Furthermore, photosynthesis is highly sensitive to abiotic stresses such as drought, high temperatures, and ozone, since they inactivate photosynthetic electron transfer and photophosphorylation, adversely affect photosynthetic metabolic processes, and lead to damage of thylakoid membranes and organelle ultrastructure (Ainsworth et al., 2013; Lobell et al., 2014; Sieber et al., 2016). In fact, an increase in atmospheric temperature can reduce crop yields by between 6 to 25% depending on the region and the crop (Sieber et al., 2016; Zhao et al., 2017). However, drought is the major abiotic stress that impairs crop production (Mishra and Cherkauer, 2010; Lobell et al., 2014; Lesk et al., 2016; Zipper et al., 2016; Matiu et al., 2017) due to photosynthetic limitations imposed by stomatal and non-stomatal processes (Tissue et al., 2005; Kohzuma et al., 2009; Dahal et al., 2014). It has been estimated that drought has caused the loss of 1820 million tons of cereal production during the last 4 decades (Lesk et al., 2016). In the future, drought occurrence and severity are projected to rise, increasing the risk of yield loss by 24% in soybean, 21% in maize, 18% in rice, and 20% in wheat (Leng and Hall, 2019). On the other hand, the predicted increase in atmospheric CO_2_ levels, as a substrate of photosynthesis, is expected to increase yields by up to 30% depending on plant species and other environmental conditions (Ainsworth and Long, 2005; Long et al., 2006; Sanz-Saez et al., 2017). It has been shown that elevated temperature and drought can negate the positive effects of elevated CO_2_ on yield (Ruiz-Vera et al., 2013; Gray et al., 2016). While plant breeders and plant biologists have worked extensively over the years to increase yields and improve plant responses to abiotic stresses, photosynthesis was often overlooked (Long et al., 2015). Advances in genomics, genetics, and modeling tools have now paved the way for improving photosynthesis to increase yields within climate change scenarios (Zhu et al., 2010; Long et al., 2015; Ort et al., 2015).

The effects of abiotic stresses on photosynthesis have given rise to numerous review articles (Hikosaka et al., 2006; Pinheiro and Chaves, 2011; Ainsworth et al., 2013; Song et al., 2014; Dusenge et al., 2019); however, many of them only focused on specific aspects. In this review, the effects of abiotic stresses are considered from a holistic point of view. It covers the use of “omics” techniques (genomics, transcriptomics, proteomics, and hormonomics) (Section “-Omics” Analyses to Identify Novel Targets and Networks Underlying the Function of the Photosynthesis Machinery: Roads to Develop Engineered Environmental Stress-Tolerant Crops Through Photosynthesis”) to identify potential target genes that could improve photosynthesis and crop yield. Whole plant physiological responses (Section “Physiological Traits Involved in the Maintenance of Photosynthesis as Tools for Crop Improvement in a Context of Climate Change”) and the development of semi- and high-throughput phenotyping techniques (Section “Semi- and High-Throughput Phenotyping Techniques to Measure Photosynthetic Traits”) are described that allow for a better understanding of major physiological traits associating the maintenance of photosynthesis with abiotic stress tolerance. To bring together the wealth of knowledge and to extrapolate the effects of the environment on photosynthetic capacity and plant development at the whole plant land canopy levels, Section “Modeling Photosynthesis in Crop Growth Models” reviews the application of photosynthetic models to calculate carbon gain for biomass production and to estimate possible future impacts of a changing climate on global crop production and grain yield. Finally, Section “Metabolic Engineering to Improve Photosynthesis and Elevated CO_2_ Acclimation” gives an overview of the application of metabolic engineering and examples of what has been successfully achieved already to improve photosynthesis and how elevated CO_2_ acclimation might limit yield improvement and quality of certain C3-plant species.

## “-Omics” Analyses to Identify Novel Targets and Networks Underlying the Function of the Photosynthesis Machinery: Roads to Develop Engineered Environmental Stress-Tolerant Crops Through Photosynthesis

**FIGURE 1:**
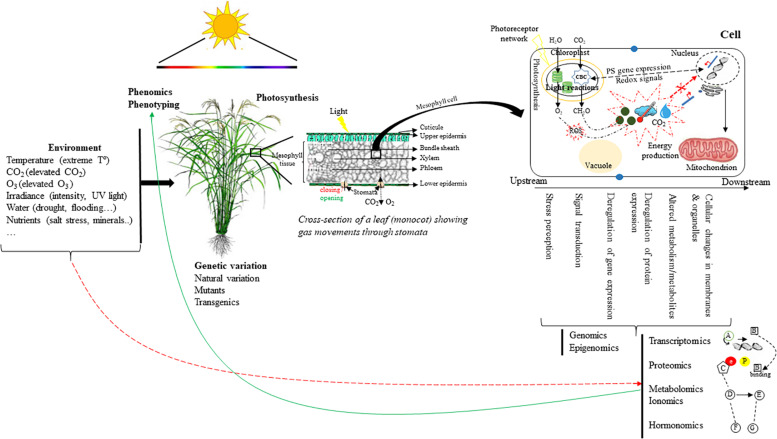
Overview of the multi-scale level concept including molecular (genetic variation, gene expression networks, proteins, metabolites, hormones, etc.), cellular, plant (organ development), and ecosystem (canopy) levels. Systems biology methods can be used to analyze and model cellular networks to obtain a simplified description of cellular functioning (scaling down). The integration of these different scales can help improve photosynthesis. In the example, rice (monocot) plants are used as a higher-scale model. Phenomics technologies allow the physiological and/or temporal phenotypic dissection of quantitative traits (i.e., carbon assimilation) controlled by a subset of target genes, proteins, metabolites and/or pathways. 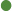 represents external and internal factors (i.e., hormones, ROS, sugars, CO_2_, T°) influencing the control of photosynthesis in plants. Red arrows show repression/activation of proteins/transcripts coordinating the expression of nuclear/plastid-encoded genes. Here, the upstream and downstream changes governing the above factors are summarized, on the one hand, by stress perception, signal transduction, and on the other hand, by genes, proteins, metabolites alterations, and reactions occurring in membranes and organelles.

The emergence of omics technologies, such as genomics, transcriptomics, proteomics, metabolomics, ionomics, and hormonomics have permitted to identify components associated with photosynthesis including molecular regulatory circuitries, photosynthetic machinery and functioning, and photoprotective mechanisms, thus underpinning factors paving the way to photosynthesis efficiency-boosting and the improved productivity and quality of modern crop varieties (Figure 1).

### Genomics to Study the Natural Variation of Plant Photosynthetic Efficiency

This section does not intend to give a detailed account of genomics and the reader is directed to other publications to read about general genomic innovation for crop improvement (Bevan et al., 2017), development of new genomic technologies (Huang et al., 2010; Takagi et al., 2013; Schlotterer et al., 2014; Varshney et al., 2014; Pandey et al., 2016; Crossa et al., 2017; Rasheed et al., 2017; Scheben et al., 2017; Watson et al., 2018), and the use of genomics in crop breeding (Varshney et al., 2012, 2018).

Evolution has been continually shaping photosynthesis, so fine-tuning this rather inefficient metabolic process could help to boost crop yields under normal and adverse conditions. This could be achieved using new plant breeding technologies to target photosynthetic processes and thus to contribute substantially to improving global food security under climate change scenarios. Conventional quantitative trait locus (QTL) mapping using recombinant inbred lines (RIL) and near-isogenic lines (NIL) is an effective tool to identify quantitative traits associated with photosynthesis and the modulation of photosynthetic parameters in response to environmental cues (Adachi et al., 2011; Gehan et al., 2015; Yan et al., 2015; de Oliveira Silva et al., 2018; Oakley et al., 2018). Indeed, RIL and NIL populations have been used to discover genetic variation and genes associated with photosynthetic efficiency, while some specific photosynthesis-related traits were found to be influenced by functional genetic variation in a limited number of genes (Oakley et al., 2018). Indeed, putative QTLs have been detected for Single-Photon Avalanche Diode (SPAD) value, chlorophyll content, stomatal conductance, sink size, source strength, carbon isotope discrimination, and carbohydrate translocation (Ulloa et al., 2000; Teng et al., 2004; Takai et al., 2010, 2013). Potential QTLs have been revealed also for net CO_2_ assimilation rate (A_n_) in rice (Ishimaru et al., 2001; Price et al., 2002; Zhao et al., 2011; Hirotsu et al., 2017; Ye et al., 2017; Adachi et al., 2019), barley (Teulat et al., 2002; Cantalapiedra et al., 2015; Liu et al., 2017; Du et al., 2019), maize (Fracheboud et al., 2002), soybean (Jun et al., 2014; Lv et al., 2018; Liu D. et al., 2019), cucumber (Zhang et al., 2004), and legumes (Muchero et al., 2009; Kumar et al., 2014; Li F. et al., 2015). In the case of rice, several loci enhancing leaf A_n_ have been detected on chromosomes 3, 4, 5, 6, 8, 9, and 11 (Adachi et al., 2011; Gu et al., 2012). In addition, some backcross inbred lines (BILs) derived from an *indica* variety, Takanari, and an elite *japonica* cultivar have 20–50% higher values of leaf A_n_ than those of the parental cultivars (Adachi et al., 2013). By using BILs and chromosome segment substitution lines (CSSLs), Adachi et al. (2019) detected 10 “qHP” (high photosynthesis) QTLs linked to an increased A_n_ during at least 2 years in the field and named qHP1a, qHP1b, qHP2, qHP3a, qHP3b, qHP4, qHP5, qHP7a, qHP7b, and qHP10. Takai et al. (2013) identified qHP4 in a chromosomal region containing the *GPS* (GREEN FOR PHOTOSYNTHESIS) gene by using the above-mentioned BIL mapping population. Similarly, a previous fine-mapping study revealed Carbon Assimilation Rate 8 (CAR8) as an A_n_-enhancing QTL (Adachi et al., 2017). Whole-genome sequencing (WGS) is another genetic tool that can be used to identify genes susceptible to make photosynthesis more efficient. This requires the development of high-resolution mapping populations in the form of genotypically detailed diversity panels suitable for genome-wide association studies (GWAS). Together, natural variation associated with different traits can be determined, thereby providing breeders with marker-trait associations that can be directly exploited for crop design (Huang and Han, 2014; Ogura and Busch, 2015; Barabaschi et al., 2016). The use of natural variation to understand the genetic basis of photosynthetic efficiency represents a powerful tool. Indeed, this approach has been widely used to reveal the genetic basis of photosynthesis-related traits under changing environmental conditions in several crops (Panthee et al., 2006; Wang et al., 2016; Lv et al., 2018). Tsai et al. (2019) investigated photosynthetic efficiency under salinity stress and identified several chromosomal regions associated with chlorophyll fluorescence parameter variations, and identified some significant SNPs linked to genes involved in salt tolerance. It has been shown also that plants exhibit genetic variation for photosynthetic response to changing irradiance levels (van Rooijen et al., 2015). Additionally, the application of GWAS as a powerful tool to identify candidate genes for the improvement of crop productivity has been validated by its role in the discovery of many genome regions and genes associated to A_n_ and chlorophyll fluorescence under different stresses (Strigens et al., 2013; Fiedler et al., 2016; Ortiz et al., 2017; Su et al., 2019). Recently, a multi-parent advanced generation intercross (MAGIC) strategy was proposed to promote genome intercrossing and shuffling (Cavanagh et al., 2008). MAGIC populations have been developed for several plant species^1^ and used to create ideotypes under climate change (Bandillo et al., 2013; Lucas et al., 2013; Muchero et al., 2013; Huynh et al., 2018).

The functional dissection of photosynthesis can be undertaken also by forward genetic screens. Strategies, identification, insights and mutant effects have been reviewed previously (Somerville, 1986; Parinov and Sundaresan, 2000; Page and Grossniklaus, 2002; Luo et al., 2018; Döring et al., 2019). Knowledge obtained from mutant screenings can reveal new chloroplast functions, including those necessary for high photosynthetic performance, and accelerate the molecular characterization required for deciphering the genetic basis of plant photosynthesis for future improvements. For instance, Döring et al. (2019) identified genomic segments that contained mutated candidate genes to create a more C4–like bundle sheath by using a mapping-by-sequencing approach. However, a successful forward genetic screen needs an easily identifiable trait followed by a validation of the identified mutated genes by state-of-the-art technologies such as T-DNA knock-out lines, RNAi lines, or by gene-editing tools (Hahn et al., 2017). Indeed, genome editing approaches, such as transcription activator-like effector nucleases (TALENs) (Bedell et al., 2012; Li et al., 2012) and the CRISPR (clustered regularly interspaced short palindromic repeats)/Cas9 RNA-guided system (Cong et al., 2013; Feng et al., 2014), will enable precise genome engineering that could be useful to improve photosynthesis by generating targeted variations for precision breeding (Scheben and Edwards, 2017; Scheben et al., 2017). Crop breeding programs will benefit from the integration of modern genomics approaches, and the use of high-throughput genotyping/phenotyping platforms (see section “Semi- and High-Throughput Phenotyping Techniques to Measure Photosynthetic Traits”). Indeed, within the context of modern plant breeding, several molecular breeding approaches have been applied to introgress genomic regions into elite lines (Varshney et al., 2012). Marker-assisted selection (MAS), marker-assisted backcrossing (MABC), and gene pyramiding programs have been widely used in crop improvement to create desirable characters including high photosynthetic efficiency under (a) biotic stress conditions (Singh and Singh, 2015; Varshney, 2016; Cobb et al., 2018). While transgenic approaches have been successful in improving plant yield through improved photosynthesis (as highlighted in section “Modeling Photosynthesis in Crop Growth Models”), the genetic mapping of desired photosynthesis-related traits will require an efficient implementation of high-throughput, non-destructive phenotyping (see section “Semi- and High-Throughput Phenotyping Techniques to Measure Photosynthetic Traits” for more details) to assess them between plant genotypes (van Bezouw et al., 2018). The gap between genomes and phenotypes will be bridged by “omics” approaches, including transcriptomics, proteomics, hormononics, and metabolomics.

### Photosynthesis and Transcriptional Regulation

**FIGURE 2:**
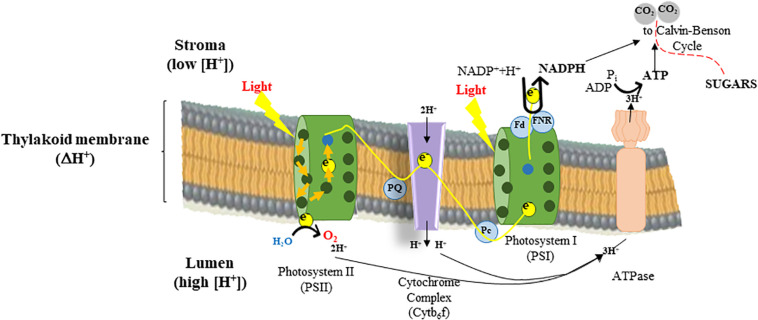
Light energy drives mainstream electron flow used by photosystem II (PSII) to reduce plastoquinone (PQ) to plastoquinol. The reducing equivalents on plastoquinol move through the electron transfer chain (ETC) to the cytochrome b_6_f complex and plastocyanin (Pc), releasing protons (H^+^) into the thylakoid lumen, while the electrons are used by photosystem I (PSI) to reduce ferredoxin (Fd), again driven by light-supported ETC through PSI. Reduced Fd is coupled to Fd:NADP(H) oxidoreductase (FNR) that catalyzes the reduction of NADP^+^ to NADPH. The oxidation of water by PSII and the oxidation of plastoquinol by the cytochrome b_6_f complex allows the generation of a ΔpH between the thylakoid lumen and the stroma, essential for generating ATP via the ATPase and balancing the proportion of ATP:NADPH produced by electron transfer that is required to function the Calvin cycle that assimilates CO_2_ and leads to sugar production, and plant growth.

About 3000 genes are required for a plant to carry out photosynthesis and high-throughput sequencing to quantify transcripts will help determine when and where a gene is turned on/off. The analysis of deregulated gene expression patterns controlling photosynthesis-related processes across a wide array of cellular responses, phenotypes, and conditions would help to engineer multiple aspects of photosynthesis in the future. This could be achieved by the manipulation of gene regulatory networks. For instance, genes encoding the four major multi-component complexes of the thylakoid membrane [PSII-LHC (light harvesting complex) II, cytb_6_f, PSI-LHCI, and ATP synthase] (*cf.* Figure 2) that work together to carry out light-dependent energy-production must be co-regulated to be efficient.

Photoreceptors regulate the expression of many genes important for plant performance including the initiation of chloroplast biogenesis, chloroplast gene transcription, chlorophyll biosynthesis and photosynthetic-associated processes like chloroplast movements and stomatal opening (Lepistö and Rintamäki, 2012; Legris et al., 2019), therefore engineering these genes could be of great interest to improve photosynthetic traits. The modulation of certain phytochrome gene families, especially PHYA and PHYB, in several crops of interest plays an important role in determining the enhancement of quality and yield as well as the development of agronomically important traits including abiotic stress tolerance (Franklin and Whitelam, 2007; Abdurakhmonov et al., 2014; Gupta et al., 2014; Gururani et al., 2015; Martín et al., 2016; Shin et al., 2016). At least 41 transcription factors (TFs) have been described in Arabidopsis to act downstream of photoreceptor genes, the most characterized being PHYTOCHROME INTERACTING FACTOR (PIF) and PIFLIKE (PIL) families of basic helix-loop-helix (bHLH) proteins. Most plastid-encoded genes appear to be regulated by several sigma factors with overlapping functions. Stress-responsive TFs such as MYC (myelocytomatosis oncogene)/MYB (myeloblastosis oncogene), bZIP, NAC (NAM, ATAF, and CUC) and ZF-HD (zinc-finger homeodomain), CBF/DREB, and AREB/ABF (ABA-responsive element-binding protein/ABA-binding factor) are known to regulate the expression of photosynthetic genes in response to abiotic stresses. Homeobox homeodomain leucine-zipper (HD-Zip) TFs have diverse functions during plant development and stress adaptation, and some members of this family are under the control of the phytochrome system such as ARABIDOPSIS THALIANA HOMEOBOX 2 (ATHB2) (Kunihiro et al., 2011). *ATHB17*-overexpressing plants enhance abiotic stress tolerance by coordinating both photosynthesis-associated nuclear genes (PhANGs) involved in the light reactions and an essential nucleus-encoded Arabidopsis σ-Like Factor (*AtSig5*) (Zhao et al., 2017). The functional analysis of transgenic wheat overexpressing Nuclear Factor Y (*TaNF-YB3*) provided evidence for the positive involvement of the TF gene *TaNF-YB3* in the regulation of photosynthesis genes leading to an increase in leaf chlorophyll content and photosynthetic rate (Stephenson et al., 2011). Rice plants over-expressing *HARDY (HRD)*, an AP2-EREF-like TF, showed drought tolerance, thicker leaves, more chloroplast-bearing mesophyll cells, and improved water use efficiency by enhancing photosynthetic assimilation and reducing transpiration thus contributing to increased biomass in a water-limiting environment (Karaba et al., 2007). Genetically engineering stress-responsive TFs regulating photosynthesis-related genes to modulate stress tolerance may hold promising beneficial traits of agronomic interest including improved productivity.

Further targets for improving photosynthetic traits could be to modulate TFs known to directly control the photosynthetic machinery. GOLDEN TWO-LIKE (GLK) TFs, key mediators of developmental control, have been implicated in positively regulating both chloroplast formation and coordinating the expression of photosynthetic apparatus genes, such as *LIGHT HARVESTING COMPLEX PROTEIN* genes and tetrapyrrole synthesis genes *HEMA1*, *GUN4*, *GUN5*/*CHLH*, and *CHLOROPHYLL A*/*B OXIDASE* (*CAO*) (Waters et al., 2009; Powell et al., 2012; Nguyen et al., 2014). Furthermore, the nuclear GATA NITRATE-INDUCIBLE CARBON-METABOLISM-INVOLVED (GNC) TF is involved in the control of both chloroplast development from the proplastid and control of chloroplast growth and division (Bastakis et al., 2018).

Since the ectopic overexpression of some genes might result in the overexpression of other genes (since many genes are coordinately regulated, for instance by photoreceptors) and increase the levels of associated proteins with undesired phenotypic modifications that could increase trade-offs within the agronomic characteristics and worsen productivity, even when photosynthetic performance has been improved. Therefore, it might be required to modulate only photosynthesis-related genes to accomplish the desired boosting of crops under adverse environmental conditions.

The physiological and biochemical changes in plants under specific stress conditions are related to altered gene expression, with a common set of about 750 nuclear-regulated genes responsive to changes in photosynthetic redox state (Jung et al., 2013). Genes showing redox-regulated expression characteristics are either directly involved in or connected to photosynthesis (Pfannschmidt, 2003). Adverse environmental conditions often lead to chloroplast damage including photoinhibition but this can be limited by acclimation mechanisms, many of which are based on ROS generation and/or the triggering of regulatory redox-reactive molecules (e.g., thioredoxins, and reduced glutathione). These redox-molecules can regulate transcription by interacting with TFs and other signaling molecules and thus deregulate the expression of photosynthetic component genes at multiple levels of signal transduction cascades and signaling pathways. Targeting such acclimation mechanisms, at the gene level, could help improve photosynthesis and plant adaptability under abiotic stresses. To achieve this, a better understanding of how triggered regulatory redox-reactive molecules deregulate the expression of photosynthetic component genes is required. Furthermore, the identification of redox signal targets and/or stress-responsive TFs could help identify unknown photosynthesis-related genes.

Although redox-associated changes in nuclear gene expression have been described, only a limited number of TFs that mediate transduction of redox signals controlling chloroplast signaling have been identified (Pesaresi et al., 2009; Petrillo et al., 2014). The over-expression of the zinc finger transcription factor, ZAT10, altered photosynthetic rates and resulted in enhanced tolerance to light and exogenous H_2_O_2_ photoinhibition, and increased expression of ROS detoxification genes whose products were targeted to multiple subcellular compartments (Rossel et al., 2007). Also, three A-type heat-shock transcription factors (HSFs) -HSFA1D, HSFA2, and HSFA3- were found to be key factors regulating the gene encoding ASCORBATE PEROXIDASE 2 (APX2) in response to a redox-generated plastid stress signal (Jung et al., 2013). Furthermore, *RADICAL INDUCED CELL DEATH PROTEIN 1 (RCD1)* stabilized the TF Rap2.4-dependent redox-regulation of genes encoding chloroplast antioxidant enzymes, although it was also found to be essential for protecting cells from photooxidative stress, in a widely redox-independent manner (Hiltscher et al., 2014). Recent promising approaches targeting chloroplast energy balance via AOX, a mitochondrial terminal alternative oxidase (Vanlerberghe, 2013; Vanlerberghe et al., 2016; Dahal and Vanlerberghe, 2017, 2018) and the overexpression of CBF (C-repeat binding factor) transcription factors (Dahal et al., 2012; Kurepin et al., 2013; Hüner et al., 2014) have been reported to enhance plant photosynthetic performance under stress conditions.

It can be seen that the manipulation of TF to modulate gene networks and gene expression is an avenue that could be exploited to engineer crops for enhanced photosynthesis and productivity under adverse environmental conditions. To achieve such aims, efforts are required to identify the appropriate TFs. Also, deep learning techniques that exploit large scale data set analyses (chromatin accessibility assays, microarray, RNA-seq expression, ChIP-seq data, gene expression profiles, DNA affinity purification sequencing, ampDAP-seq) to help resolve complex biological problems in transcriptomics need to be developed further.

### Proteomics

Photosynthesis is mediated by the coordinated action of ca. 3000 nuclear-encoded preproteins synthesized in the cytosol and imported into organelles through special machineries (Nakai, 2015; Baslam et al., 2016) in envelope membranes. About 2400 of these proteins are found in the chloroplast (Friso et al., 2004), while only ca. 100 proteins are encoded by the chloroplast genome. Many environmental changes lead to an imbalance in photosynthetic electron transfer due to a modification of the redox potential of ETC components as well as functionally coupled pools of redox-active compounds (e.g., thioredoxins and glutathiones) thus affecting photosynthetic efficiency (Roach and Krieger-Liszkay, 2014; Larosa et al., 2018; Jimbo et al., 2019; Takagi et al., 2019). This imbalance can be redressed by the photosynthetic control of LHC, PS, and cytb_6_f stoichiometry.

In order to prevent ROS generation, PSI must be induced to accept electrons when PSII is strongly active in the daytime by shorter-wavelength light. The mechanism of induction of PSI occurs through the de-phosphorylation of sigma factors by redox signals monitoring PQ status (Shimizu et al., 2010). Redox proteomics has been developed to monitor the redox dynamics of cellular proteins under environmental stimuli (Ansong et al., 2014; Sadler et al., 2014; Ameztoy et al., 2019). Application of this technology to plants and chloroplasts has identified novel protein targets undergoing thiol modifications [e.g., NADPH-dependent thioredoxin reductase C (NTRC), chloroplastic fructose 1,6-bisphosphatase (FBPase)] and plastid redox signaling networks to maintain a high photosynthesis efficiency which is important for the global adjustment of plant metabolism (Lindahl and Kieselbach, 2009; Hall et al., 2010; Dietz and Pfannschmidt, 2011; Ameztoy et al., 2019). Quantitative phosphoproteomic profiling using isobaric tags for relative and absolute quantitation (iTRAQ) showed that ROS generated by an oxidative burst under drought stress could trigger NO synthesis to protect the photosynthetic apparatus by modulating the phosphorylation of diverse proteins such as LHC, thylakoid-bound Ser/Thr kinase STN7, and chloroplast post-illumination chlorophyll-fluorescence-increase protein (PIFI). ROS produced under drought conditions provoked an increase of the cellular concentration of Fe^2+^ ions, resulting in an increased electron transfer to oxygen *via* the Fenton reaction (de Carvalho, 2008). Similar effects are observed under nutrient starvation, including Mg^2+^ and Fe^2+^, which are essential co-factors for several redox-active proteins in the photosynthetic ETC. More recently, Inomata et al. (2018) reported the chloroplast phosphoproteome profile of a rice *nucleotide pyrophosphatase*/*phosphodiesterase 1 (NPP1)* mutant. This study highlighted that the loss-of-function of NPP1 in rice leaves increased stomatal conductance, photosynthesis, starch, and sucrose storage while also impacting proteins involved in carbohydrate metabolism and protein synthesis system under high temperature and CO_2_ conditions. Their data indicated that NPP1 plays a crucial role in carbon flux by transporting carbon taken up from starch and from cell wall polysaccharide biosynthesis to other metabolic pathways in response to the physiological needs of the cell.

Using proteomics, five new photosynthetic activity responsive transcriptional regulators were classed as redox-active in response to nutrient limitation in the photosynthetic cyanobacteria *Synechococcus* sp. PCC 7002. These were RbcR regulating the rbc LXS operon, Fur and Zur regulating iron and zinc homeostasis, respectively, cyAbrB regulating N and C metabolisms, and a TetR family regulator (Sadler et al., 2014). Furthermore, proteomics has led to the identification of proteins that mediate redox control during RNA maturation and transcription. These RNA plastid-encoded polymerase (PEP)-associated proteins are plastid transcription kinases (PTKs) (such as STN, CSK, and cpCK2), which respond to changes in thiol/disulfide redox state mediated by glutathione (Baginsky et al., 1999), and can phosphorylate the sigma-like TF, *SIG6*, involved in the regulation of chloroplast gene transcription. Similarly, these PTKs are under the control of the chloroplast GSH (glutathione) pool, suggesting a GSH-mediating redox control of their activities (Baena-González et al., 2002). Proteomics has identified also several heat-responsive TFs and proteins, such as MYB, WRKY, DnaJ protein (LeCDJ1), heat shock proteins (HSPs), filamentous temperature-sensitive H (ftsH11), sedoheptulose-1,7-bisphosphatase (SBPase), and constitutive or stress-inducible key enzymes (Chen et al., 2006; Rushton et al., 2012; Yang et al., 2012; Grover et al., 2013; Kong et al., 2014). (Phospho)-proteomic analyses suggested that heat-responsive phosphorylation levels of some important proteins [e.g., protochlorophyllide reductase (POR), oxygen-evolving complex (OEC), RuBisCO, and phosphoenolpyruvate carboxykinase (PEPCK)] were modulated, thus indicating that post-translational modifications (PTMs) were critical processes for plant heat tolerance (Hu et al., 2015; Walker et al., 2016). A proteomic approach has shown the role of PSII protein phosphorylation [e.g., the minor antenna polypeptide Lhcb4 (CP29)] in PSII protection and in the photoinhibition-repair cycle (reviewed in Aro et al., 2004).

In order to optimize leaf gas exchange under stressful environmental conditions, proteins related to stomatal development have been identified. Indeed, plants can modulate stomatal aperture, density, and placement through signaling pathways involving peptide ligands, transmembrane receptors, and mitogen-activated protein kinase (MAPK) modules. The TFs bHLH [including both MUTE and FAMA, inducer of CBF expression 1 (ICE1/SCRMI), HIGH CARBON DIOXIDE (HIC) protein, PHYTOCROME INTERACTING FACTOR (PIF), mitogen-activated protein kinases (MPKs), and their upstream MKK, YODA, SPCH, C2/H2-type zinc-finger proteins (SZT and AZF2)] have been described to regulate stomatal response to environmental perturbations and improve stress tolerance (Gray et al., 2000; Chinnusamy et al., 2003; Sakamoto et al., 2004; MacAlister et al., 2007; Wang et al., 2007; Kanaoka et al., 2008; Casson et al., 2009; Pillitteri and Dong, 2013). EPIDERMAL PATTERNING FACTOR 1 and 2, and STOMAGEN are secreted peptides that regulate the function and development of stomata (Hara et al., 2007; Hunt and Gray, 2009; Sugano et al., 2010). Furthermore, the α-subunit of the heterodimeric G protein (GPA1) and ERECTA protein are known to regulate plant transpiration efficiency by regulating stomatal density (Masle et al., 2005).

Interestingly, chloroplast proteome turnover is crucial to cell homeostasis and adaptation to changing conditions. In their review, Izumi and Nakamura analyzed the influence of extra-plastidial processes on the turnover of chloroplast proteins (Izumi and Nakamura, 2018). Fine-tuning protein turnover, and/or increasing the efficiency of respiratory ATP production can help “maintenance respiration” -the energy required to maintain mature tissue biomass when growth rate is zero (Thornley, 2011; O’Leary et al., 2017; Machacova et al., 2019)-, and hence reduce carbon loss. This process can be a primordial factor in determining growth rate and it may also impact biomass formation. Indeed, growth rate is negatively correlated with protein turnover among Arabidopsis accessions (Ishihara et al., 2017). For instance, eliminating THI4 (a suicide enzyme in thiamin biosynthesis) protein turnover, increased crop biomass accumulation by 4% by essentially reducing the high energy costs and loss of photosynthetically fixed carbon to produce thiamin (Hanson et al., 2018).

As thousands of different proteins make up the machinery of plant cells, proteomics and its derivatives (phosphoproteomics, redox proteomics, and peptidomics) are important tools to better understand processes that regulate protein synthesis and degradation in plants such as protein turnover, abundance, location, compartment-specific proteases/peptidases, protein interactors, and PTMs (e.g., phosphorylation, ubiquitination, nitrosylation, and carbonylation) in steady and non-steady state scenarios. Establishing an integrated understanding of the processes that underpin changes in protein expression under several physiological and developmental scenarios could define new targets to rationally engineer photosynthesis for agronomic gain.

### Hormonomics

Chloroplasts synthesize hormones that are known to play a critical role in photosynthesis gene expression and to participate as signaling molecules in stress signal transduction. Phytohormones including brassinosteroids (BRs), abscisic acid (ABA), cytokinins (CKs), salicylic acid (SA), ethylene, jasmonate, and auxins have been implicated in the control of stomatal development and function in response to environmental stresses, which ultimately impact photosynthesis. The importance of ABA as a central regulator and integrator of long-term changes in stomatal behavior was revealed by Dittrich et al. (2019). Under stress environments, such as drought, ABA induces stomatal closure through calcium-sensing receptor signaling driven by NO accumulation via H_2_O_2_ production in guard cell chloroplasts leading to membrane depolarization and loss of guard cell volume and turgor (Wang et al., 2012). ABA-dependent guard cell closure has been shown also to be regulated by the guard cell anion channel SLAC1, together with the protein kinase OST1 (Hedrich and Geiger, 2017). Using genetic approaches, Chater et al. (2015) showed that either guard cell ABA or ABA receptors, PYR/PYL/RCAR, were sufficient to mediate a [CO_2_]-induced stomatal density response. However, recently a model for the convergence of CO_2_ and ABA signal transduction downstream of OST1 protein kinase activation has been reported (Hsu et al., 2018). Transgenic rice and Arabidopsis plants overexpressing the pyrabactin resistance 1-like (*PYL*) family of ABA receptors promoted resistance to extreme drought stress by reducing transpiration rate and stomatal conductance thus enhancing the photosynthetic rate and water use efficiency (Zhao et al., 2016). Efforts have been made to improve photosynthetic efficiency by engineering the photosynthesis-related transcription factor, ABA-responsive 17 (*ABR17*) (Grover et al., 2013). Constitutive expression of ABA-responsive element-binding protein (*ABP9*) increased photosynthetic capacity, carbon use efficiency and tolerance to high temperature and water stress (Zhang et al., 2008). Xiong and Zhu (2003) suggested that genotypes with putative constitutive high ABA concentrations could be more tolerant to environmental stresses. ABA can also protect the photosynthetic apparatus against photoinhibition by modulating the xanthophyll cycle and by increasing the recovery rate of photo-inactivated PSII complexes (Saradhi et al., 2000). Therefore, altering stomatal sensitivity to ABA could allow plant acclimation to changing environments by optimizing gas exchange for photosynthesis.

A water deficit stimulates not only ABA synthesis but inhibits the production of CKs resulting in an imbalance between the two hormones in leaf tissues and this can control physiological responses (e.g., stomatal closure) that lead to whole plant higher adaptive fitness (Pospisilova et al., 2005; Tanaka et al., 2006). The action of CKs is mediated mainly by AHK3 receptors and several TFs (i.e., *ARR1*, *ARR10*, and *ARR12*) that regulate nuclear gene expression encoding plastid proteins (e.g., LHC, RuBisCO), plastid-related protein abundance [e.g., gamma-subunit of ATP synthase, glyceraldehyde-3-phosphate dehydrogenase (GAPDH), ClpP, ribosomal protein L21], and downstream TFs (i.e., *CGA1*, *GNC*, *HY5*, *GLK2*, *CRF2*). In this way, CKs modulate chloroplast development, division, and function (Chiang et al., 2012; Cortleven and Schmülling, 2015; Okazaki et al., 2015). Transcriptomic responses to CKs include over 100 different photosynthesis genes (Brenner et al., 2012) while a (phospho)-proteomic study identified about 50% of CK-regulated proteins to be localized in the chloroplast (Černý et al., 2011). Under high light stress, CKs show a protective function by decreasing photoinhibition, mediated by AHK2 and AHK3 receptors and the TFs ARR1 and ARR12 (Cortleven et al., 2014).

Genetic and pharmacological studies have implicated BRs in stomatal development and patterning. Kim et al. (2012) reported that the BR-insensitive mutants *bri1-116* and *bsu-q* (*amiRNA-BSL2,3 bsu1 bsl1* quadruple mutant) contained only paired guard cells, and lacked other epidermal cells. Genetic analyses indicated that receptor kinase-mediated BR signaling inhibited stomatal development through glycogen synthase kinase 3 (GSK3)-like kinase BIN2, which acts upstream of the MAPKKK YODA, and mediates signaling by ERECTA family receptor kinases. Previous studies had also demonstrated key functions of BRs in inhibiting photosynthetic gene expression, and promoting cell elongation, chloroplast senescence, and floral induction (Li et al., 1996). Furthermore, it was found in leaves and cotyledons that BR-promoted stomatal formation was via a cross-communication of the YDA-MKK4/5-MPK3/6 cascade and the basic helix-loop-helix transcription factor SPEECHLESS (SPCH), a regulator of the entry, amplifying and spacing divisions that occur during stomatal lineage development (Gudesblat et al., 2012).

As photosynthetic gas exchange and transpiration balance are impacted by altered stomatal patterning under changing environmental conditions, auxin control of stem cell compartment size, as well as auxin depletion as the switch from unequal to equal divisions, play key roles during stomatal development. High auxin activity has been observed during unequal cell divisions in stomatal patterning, whereas a decrease in auxin activity promoted guard mother cell (GMC) fate and its subsequent equal division into two guard cells. Similarly, an auxin-resistant mutant where AUX/IAA proteins failed to interact with the auxin receptor, leading to auxin insensitivity, was defective in the suppression of stomatal development in dark-grown seedlings (Balcerowicz et al., 2014). Zhang et al. (2014) reported that auxin negatively regulated stomatal development through MONOPTEROS (MP) repression of mobile peptide STOMAGEN gene expression in mesophyll cells where photosynthesis mainly takes place (Zhang et al., 2014). The regulation of stomatal and vascular developments by MP indicated that MP should play a role in photosynthesis and the transpiration system for optimizing plant growth and development. Loss-of-function quadruple mutants, *pin2, 3, 4, 7* and *pin1, 3, 4, 7* of the PIN gene family, controlling PIN protein-mediated auxin transport, showed stomatal defects (Le et al., 2014). Moreover, Ogura et al. (2019) identified a new gene, *EXOCYST70A3* that directly regulated root system architecture by affecting the distribution of PIN4 and hence controlling the auxin pathway without disrupting other pathways. This study suggested that EXO70A3-dependent variation in the control of root system architecture could result in improved photosynthesis and help plants fight climate change. Taken together, such studies showed the important roles of stomata in photosynthesis and global carbon and water circulation and suggest that coordinating stomatal development with photosynthesis could be achieved by manipulating auxin signaling specifically in the mesophyll cells without disturbing whole plant development.

Salicylic acid (SA) acts as an important signaling molecule and influences various physiological and biochemical functions in plants, playing an important role in plant responses to biotic and abiotic stresses. Under chilling stress conditions, inhibition of SA biosynthesis by L-α-aminooxy-β-phenyl propionic acid (AOPP) increased PSII photooxidation, leading to the generation of ROS and impairment of photosynthesis and growth, whereas applying SA at moderate concentrations induced a stress tolerance by restoring the photosynthetic machinery (Cheng et al., 2016). Other studies have shown that SA treatment alleviated carbon assimilation and several components of PSII electron transfer under heat stress by increasing proline production through the increase in γ-glutamyl kinase (GK) and a decrease in proline oxidase (PROX) activity, resulting in the promotion of both osmotic and water potentials necessary for maintaining photosynthetic activity (Wang et al., 2010; Nazar et al., 2011). Under salt stress, it was revealed that SA could modulate photosynthetic capacity due to its interaction with metabolic signaling by ROS (including H_2_O_2_), and glutathione (Arfan et al., 2007; Nazar et al., 2011; Sewelam et al., 2016). Indeed, Miura et al. (2013) reported that SA accumulation in *siz1* [small ubiquitin-like modifier (SUMO) E3 ligase] mutant plants enhanced stomatal closure and drought tolerance by controlling guard cell ROS accumulation, while the introduction of salicylate hydroxylase (*NAHG*) into *siz1*, which reduced SA accumulation, restored stomatal opening (Miura et al., 2013). Furthermore, other SA-accumulating mutants, *cpr5* and *acd6*, exhibited stomatal closure thus reducing the entry of sufficient CO_2_ for optimal photosynthesis while hindering the movement of water vapor and hence leading to drought tolerance (Miura et al., 2013).

In addition to the SA pathway, jasmonic acid (JA)-signaling (co)-regulates a wide-range of plant developmental processes and responses to biotic and abiotic stresses that probably involve the photosynthesis machinery. Indeed, the examination of high-throughput gene expression data for heat stress and methyl jasmonate (MeJA) responsive genes using GENEVESTIGATOR (Zimmermann et al., 2004), an online tool for large-scale expression data analysis, revealed a preponderance of genes associated with protein translation and photosynthetic electron transport, which could represent features associated with cellular recovery following heat stress (Clarke et al., 2009).

Ethylene receptor mutants show altered photosynthetic properties and they are sensitive to abiotic stresses. Indeed, Arabidopsis *etr1* mutants have demonstrated the role of ethylene receptor ETR1 in guard cell H_2_O_2_ signaling (Desikan et al., 2005). Other studies showed that ethylene-insensitive mutants, *etr1-1* and *ein2*, had smaller stomata, possessed lower chlorophyll and CAB (chlorophyll a/b binding complex) contents, RuBisCO activities, and had a lower whole-plant and leaf photosynthetic capacity, suggesting the role of basal ethylene perception in controlling stomatal conductance and photosynthetic capacity (Grbic and Bleecker, 1995; Tholen et al., 2007). Other seminal works suggested that ethylene-responsiveness was required for the fine regulation of PSII photochemical efficiency (Kim et al., 2017) and carbon fixation by achieving maximal RuBisCO activities through ethylene-responsive factors (ERFs) (Bracher et al., 2017; Xie et al., 2017). The control of photosynthesis by ethylene also affected plant biomass production by influencing final plant size (Ceusters and Van de Poel, 2018). Ethylene was found to directly control photosynthesis in juvenile non-senescing leaves and acted indirectly in mature leaves by promoting senescence.

In conclusion, it can be seen that hormonal networks influence plant photosynthesis and therefore they could assist us to develop new strategies to improve plant productivity and to help plants tolerate severe environmental conditions.

## Physiological Traits Involved in the Maintenance of Photosynthesis as Tools for Crop Improvement in a Context of Climate Change

Crop growth is linked to the assimilation of ambient CO_2_ through photosynthesis, in which green plants convert sunlight, water, and CO_2_ into O_2_ and carbohydrates. During the last decade, different studies have highlighted that the improvement of plant photosynthetic rates can be a strategic tool to increase crop yields (Reynolds et al., 2011). Several studies analyzing the impact of overexpression of proteins linked with CO_2_ assimilation have shown an increase in photosynthesis and plant growth (Driever et al., 2017; Kubis and Bar-Even, 2019; Ermakova et al., 2019; see section “Metabolic Engineering to Improve Photosynthesis and Elevated CO_2_ Acclimation” for details). Further, as described by Parry et al. (2011), increases in wheat yield potential during the last decades have been associated with increased photosynthesis while Flood et al. (2011) have shown that variations in either the efficiency or capacity of photosynthesis can lead to variations in growth rate and productivity. Within this context, the adaptive potential of photosynthesis to changing environments depends on the degree of genetic variation for photosynthesis that is present within a population (Flood et al., 2011). Indeed, different studies (Peng et al., 2001; Hubbart et al., 2007) show that since 1980, increases in rice yield, rather than harvest index, correlate better with increases in biomass. Furthermore, the fact that varieties released after the 1980’s show higher saturating photosynthetic rates when compared to older varieties suggest that varieties with higher biomass values would be the ones with improved photosynthesis. This suggests that breeding programs aiming to improve crop biomass production will also have an effect on photosynthetic physiology (Flood et al., 2011). Supporting this observation, the increase in crop yields detected in plants grown under elevated [CO_2_] (Ainsworth and Long, 2005; Long et al., 2006; Sanz-Saez et al., 2017; Torralbo et al., 2019) are also associated with higher photosynthetic rates measured under such conditions.

Yield depends on many factors such as the efficiency of light interception (LI), the radiation use efficiency of light energy conversion to biomass (RUE) and the fraction of biomass that is contained in harvested organs. Leaf morphological and physiological characteristics are two target factors conditioning variation in photosynthetic properties of individual leaves that are influenced by environment and genetics (Flood et al., 2011). Furthermore, genetically based differences in leaf morphology are commonly encountered at the interspecific level, and often correlate with growth (Hikosaka, 2010). During the last decade, the enhancement of plant light capturing surface and conversion of light energy has been a major target of crop breeders (Murchie et al., 2009). Within this context, a clear example of this strategy has been the increase in the development of erect leaves with a higher leaf area per unit ground area that enables more efficient radiation capture (Murchie et al., 2009). Despite this, it should be noted that the major step that is not yet near to the maximum is light conversion efficiency to biomass which is only at 50% of its theoretical level (see Zhu et al., 2008; Long et al., 2015; Slattery and Ort, 2015). However, despite its potential, selection based on improving photosynthesis was not properly considered during the last decades.

The assimilation of CO_2_ is a complex process that involves multiple genes, regulatory mechanisms, and different metabolic pathways and plant structures working together. The overall photosynthetic process is determined by CO_2_ diffusion to the chloroplast (conditioned by stomatal opening and mesophyll conductance), the capture and conversion of light energy to make ATP and NADPH (the light reactions) required for the assimilation of CO_2_ to produce sugar-phosphates used to regenerate RuBP, the molecule used to fix CO_2_ by RuBisCO, and to produce complex sugars like starch and sucrose. However, as mentioned in the introduction, O_2_ competes with CO_2_ at the RuBisCO active site thus reducing photosynthetic CO_2_ assimilation capacity and producing toxic 2-PG (Flugel et al., 2017) that is removed by the photorespiratory cycle. Photorespiration has a high energetic cost and it leads to the potential loss of carbon and nitrogen in the form of CO_2_ and ammonium. It has been calculated that photorespiration can reduce photosynthetic energy conversion to yield of certain important C3 grain plants by 20−50% (see South et al., 2019), including soybean and wheat (Walker et al., 2016). Therefore, photorespiration became a target for crop improvement (see section “Metabolic Engineering to Improve Photosynthesis and Elevated CO_2_ Acclimation”). However recent studies (Betti et al., 2016; Eisenhut et al., 2017) suggest that reducing photorespiration may not always have beneficial effects since a higher photorespiratory capacity would contribute to: (1) maintaining Calvin cycle activity; (2) decreasing excess reducing power (a target under stressful growth conditions such as exposure to drought, salinity, cold, etc.); (3) improving nitrate assimilation under elevated CO_2_ conditions. Similarly, it was found that under low CO_2_ availability conditions, unrestricted photorespiratory metabolism favored plant performance (Eisenhut et al., 2017). Therefore, modulating photorespiration would probably be important to maintain or improve crop yield under certain environmental conditions that alter the chloroplast CO_2_/O_2_ ratio in favor of O_2_.

## Semi- and High-Throughput Phenotyping Techniques to Measure Photosynthetic Traits

Within the context of climate change, it is crucial to identify the crops that will perform better under the current and near-future conditions in the field. However, current breeding programs are constrained by the limitations of field phenotyping methods (Araus et al., 2018). During the last decade, different phenotyping platforms have emerged as a strategic tool to characterize crop performance. The light reactions can be studied by measuring chlorophyll fluorescence, whereas photosynthesis and respiration are studied by measuring CO_2_ exchange between the plant and the atmosphere using infrared gas analyzers (IRGA). Depending on the type of parameter, measurements can take a few minutes, such as leaf chlorophyll fluorescence or respiration measurements, to 30−90 min, as is the case of photosynthetic parameters such as maximum rate of RuBisCO carboxylation (V_cmax_) and maximal rate of electron transport (J_max_) that are calculated using photosynthesis to CO_2_ curves, named A-Ci curves (Farquhar et al., 1980; Bernacchi et al., 2003).

These parameters can be used to distinguish differences of photosynthetic efficiency under different environments allowing researchers to identify better-adapted cultivars (Aranjuelo et al., 2009, 2013; Sanz-Saez et al., 2017); or be used as input parameters for earth systems models that predict ecosystem responses to environmental changes (Rogers, 2014). However, a lack of information about V_cmax_ and J_max_ in some species in several ecosystems is the major source of error using earth systems models (Rogers, 2014). Another parameter that can be useful for the selection of abiotic stress-tolerant cultivars is dark respiration (R_d_) (Vanlerberghe and McIntosh, 1997; Millar et al., 2011). Recently, high-throughput methodologies based on O_2_ consumption have been developed (O’Leary et al., 2017; Scafaro et al., 2017), and they can rapidly (in 1−2 min) measure precise respiration rates. However, this requires the leaf to be removed from the plant and introduced into a measuring chamber, therefore it is destructive and thus not the best option. The latest technology used to estimate this parameter is a non-destructive technique that uses leaf reflectance spectroscopy, and it will be described below.

With the rise of the genomic era, screening of entire populations for traits of interest has become paramount to associate specific genomic regions with a given plant trait (see Section “Genomics to Study the Natural Variation of Plant Photosynthetic Efficiency”). Genomic approaches need the implementation of technologies that allow the rapid measurement of photosynthetic and fluorescence traits to screen hundreds of cultivars in the shortest amount of time. Here, we will summarize semi- and high-throughput phenotyping methods to estimate parameters related to: (1) gas exchange such as V_c__max_, J_max_, and R_d_ using the latest LI-COR 6400 and LI-COR 6800 methodologies as well as hyperspectral reflectance; and (2) chlorophyll fluorescence such as solar-induced fluorescence (SIF) and stimulated fluorescence by a known source of light.

### Semi- and High-Throughput Phenotyping Methods Related to Gas Exchange Parameters

In this subsection, the most recent literature focusing on two aspects of high-throughput phenotyping (HTP) of photosynthetic parameters are summarized and discussed: (1) New semi-HTP methodologies to estimate V_c__max_ and J_max_ using the Rapid A-Ci Response (RACiR) method for LICOR IRGA equipment and the use of the leaf excision method to estimate V_cmax_, J_max_, and light-saturated photosynthesis. (2) The use of hyperspectral reflectance technology to estimate gas exchange parameters such as V_c__max_, J_max_, and R_d_.

#### Semi High Throughput Phenotyping Methods to Measure Gas Exchange Parameters

In order to estimate V_cmax_ and J_max_, A-Ci curves need to be performed using an IRGA system. In regular A-Ci curves, the leaf receives different CO_2_ concentrations ([CO_2_]) in the IRGA chamber containing the leaf, usually from 50 μmol CO_2_ mol^–1^ up to 2000 μmol CO_2_ mol^–1^ (Long and Bernacchi, 2003). During this measurement, each time that [CO_2_] is increased, leaf photosynthesis and stomatal conductance are measured after reaching a steady-state equilibrium (Long and Bernacchi, 2003), which may take between 3 and 6 min per step. In this way, 30 to 90 min are needed per one A-Ci curve, which makes this method a Low Throughput Phenotyping technique.

**FIGURE 3:**
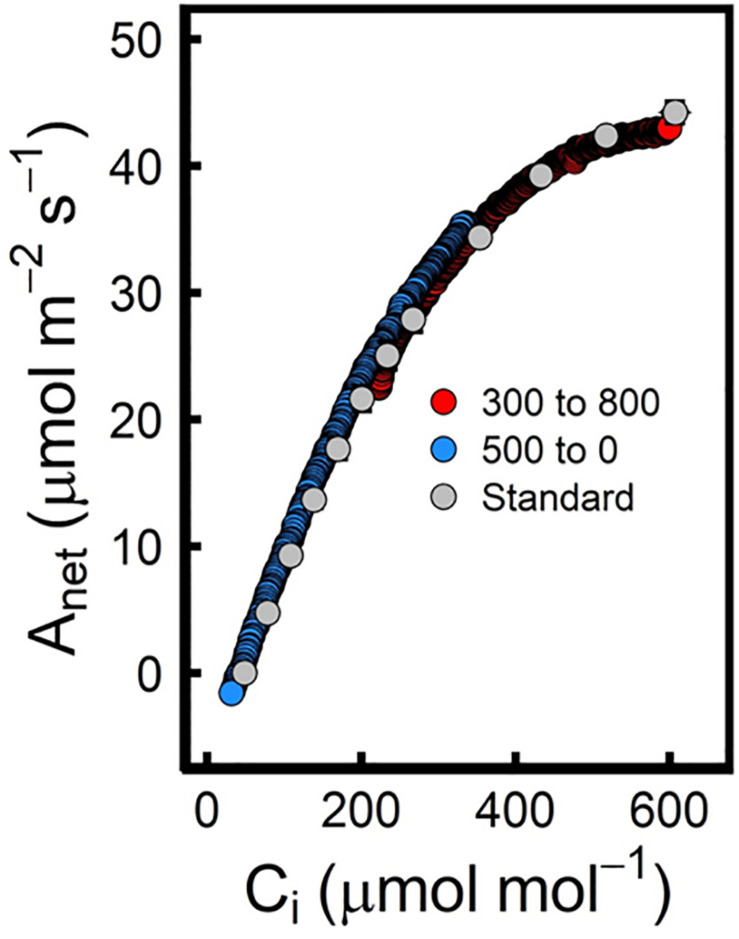
The Rapid A-Ci curve method generates a wealth of data that when corrected to reduce the lags of the empty chamber, etc., overlay well onto the “standard” A-Ci curve. In this case, a modification of an A-Ci curve from Populus deltoides is shown. The graph has been adapted from Figure 2 (Stinziano et al., 2017) by the author of the manuscript. Data for the “standard” A–Ci curve are presented as the mean of the mean (s.e.m.) for three measured responses on the same leaf of a single seedling (gray dots), while RACiRs were replicated once per CO_2_ range per seedling on the same leaf at the same location as the “standard” A–Ci curve. The best RACiRs were produced by ramping [CO_2_] from 500 to 0 (blue) and from 300 to 800 (red). For statistical information about the curve fits and the calculated parameters, please check Stinziano et al. (2017).

Due to modifications in the way that the reference and sample IRGAs are placed in the new LI6800, Stinziano et al. (2017) were able to develop a Rapid A-Ci response curve protocol with a duration of approximately 12 min. The new design can minimize lags between the reference and the sample IRGAs thus generating a constant ramp rate for CO_2_ control. In this method, the leaf is first stabilized at a [CO_2_] of 500 μmol mol^–1^ before being reduced to 0 μmol mol^–1^ at a rate of 100 μmol mol^–1^ min^–1^. Data is recorded at a rate of 0.5 Hz, which is equivalent to a measurement every 2 s, therefore assuring that changes in photosynthetic response can be recorded. In order not to miss data near the inflection point of the A-Ci response curve, Stinziano et al. (2017) added another set of measurements from 300 μmol mol^–1^ to 800 μmol mol^–1^ to complete the curve (Figure 3). Plotting together these 2 curves, the authors were able to fit the data to the Farquhar-von Caemmerer-Berry (FvCB) model, thus obtaining V_c__max_ and J_max_ estimates that were very close to those calculated from a standard A-Ci curve. However, this method has some limitations; for example, although the physical separation between the reference and the sample chamber has been reduced, it still produces a lag between the two signals that is increased when the volume of the sample chamber has to be mixed. This lag creates a differential in CO_2_ concentration that if not corrected can cause very significant variations in the measurements. To correct this lag, an empty chamber rapid A-Ci curve is run for each CO_2_ ramp (Stinziano et al., 2017). In addition, Stinziano et al. (2019) produced a best practices guide in which they indicated under which conditions an empty chamber A-Ci curve was needed. Taylor and Long (2019) found significant offsets in R_d_ (95% variation) and CO_2_ compensation point (Γ, 11% variation). According to their published data, RACiR curves can be a good tool to perform semi-HTP measurements in plant populations, being able to perform up to 60−80 A-Ci curves per day (8-h day) and per machine. However, when starting any experiment, a set of standard A-Ci curves should be performed to test that the method is working for each species and/or environmental condition. Therefore, this RACiR methodology only appears to be worth the effort when analyzing hundreds of samples at the same environmental condition as is often the case for GWAS and/or QTL experiments (Dhanapal et al., 2015; Herritt et al., 2018; Luo et al., 2018). For small experiments where only a few cultivars/species are to be analyzed, it is more reasonable to do standard A-Ci curve measurements even if it is more time consuming, as they can be used to obtain other important information such as C_c_, g_m_ (Harley et al., 1992), R_d_ and Γ that can give further valuable information about the physiological state of the plant.

Other problems occurring when measuring A-Ci curves or mid-day photosynthesis under field conditions on hundreds of samples include a transient decrease in water potential, a decrease in chloroplast inorganic phosphate concentration, and a decrease in maximum PSII efficiency. These can all occur after a few hours of light exposure making it difficult to compare measurements at the beginning with those taken at the end of the day (Ainsworth et al., 2004). When measurements are performed in the field, changing environmental conditions can alter the photosynthetic response of the plants thus making it difficult to determine treatment effects. With this in mind, Ainsworth et al. (2004) developed the “leave scission method” where soybean leaves were cut pre-dawn under water, stored in the dark, and stimulated at saturating light, at least 30 min before measurements were recorded. Using this method, all samples were measured under the same temperature, light intensity, and biochemical state. A-Ci curves have been performed in this way for the last 15 years with successful results with soybean (Ainsworth et al., 2004; Bernacchi et al., 2005; Sanz-Saez et al., 2015, 2017) and corn (Leakey et al., 2006; Yendrek et al., 2017). Additionally, Choquette et al. (2019) used this methodology to phenotype light-saturated photosynthesis response to elevated ozone in a panel of 48 corn lines, measuring more than 200 plots per day. Although this technique cannot be considered high-throughput, we believe that it could be used to screen photosynthetic parameters in diversity panel populations of about 200 lines for several days to cover the different replications. The fact that all measurements are taken under the same conditions reduces the variability associated with weather changes that happen during sampling and allows differentiating between treatments and cultivars (Choquette et al., 2019).

#### Use of Hyperspectral Reflectance Technology to Estimate Gas Exchange Parameters

Hyperspectral sensors capture electromagnetic radiation reflected from vegetation in the visible (VIS, 400–700 nm), near-infrared (NIR, 700–1300 nm), and short-wavelength infrared regions (SWIR, 1400–3000 nm), which contain information about leaf physiological status, including pigments, structural constituents of biomass, and water content (Curran, 1989; Penuelas and Filella, 1998). Variation of foliar reflectance at different wavelengths is specific to variations in the chemical and structural characteristics of the leaf (Serbin et al., 2012). With the improvement of computational methods, predictive models using partial-least square regression (PLSR) have been used to create equations that predict other physiological parameters such as leaf isotopic ratio (Richardson and Reeves, 2005), specific leaf area (Asner and Martin, 2008; Asner et al., 2011), leaf carbohydrate content (Dreccer et al., 2014; Asner and Martin, 2015), and leaf mineral content (Mahajan et al., 2014). The use of hyperspectral reflectance spectroscopy as a HTP tool has been recognized as promising in agricultural research (Weber et al., 2012; Araus and Cairns, 2014), however, until recently its utility to differentiate between a big set of cultivars had not been tested. We will focus now on the methodologies used to produce models capable of predicting photosynthetic parameters such as V_c__max_ and J_max_ and R_d_.

As measuring A-Ci curves is a tedious technique to calculate V_c__max_ and J_max_, Serbin et al. (2012) tested the possibility to predict these parameters using hyperspectral reflectance and PLSR models in aspen and cottonwood seedlings grown at different atmospheric temperatures. Because of large phenotypic variations in V_c__max_ and J_max_ due to the temperature treatments and the inclusion of two different species, the correlation between the predicted data using hyperspectral reflectance measurements and the standard A-Ci curves was very high (R^2^ of 0.89 and 0.93, respectively). This breakthrough publication demonstrated that it was possible to use hyperspectral reflectance data to estimate photosynthetic parameters. Following this discovery, Ainsworth et al. (2014) carried out a similar experiment with soybean grown at ambient and elevated ozone (O_3_) in which standard A-Ci curves were combined with hyperspectral reflectance measurements using the Field Spec Hi-Res 4 (ASD technologies). Although the number of samples was not very high (59), the phenotypic variation due to the O_3_ treatment resulted in a good correlation between the predicted and the standard A-Ci curve (R^2^ of 0.88). More recently, similar correlations between standard and predicted V_cmax_ estimations have been found for corn (Yendrek et al., 2017; R^2^ of 0.6) and wheat (Silva-Perez et al., 2018; R^2^ of 0.62).

These results are very promising for applications using very big sample sets however, to date, nobody has applied this technique to estimate V_c__max_ without testing its accuracy with standard A-Ci curve measurements. Although it seems risky, this is the avenue to take if we want to increase the speed of analysis and contribute to future breeding. To break this barrier, Choquette et al. (2019) tested 45 F_1_ corn hybrids with a differential response to elevated O_3_ under field atmospheric conditions. The effect of elevated O_3_ was studied by performing photosynthesis measurements under light-saturating conditions using a LI6400, and estimations of V_c__max_ using hyperspectral reflectance data and equations developed by Yendrek et al. (2017). In this way, Choquette et al. (2019) showed that they could detect both genotypic and O_3_ effects on predicted V_c__max_ using hyperspectral data. They found good correlations between V_c__max_ and other variables estimated using the spectra such as chlorophyll content, a parameter that had a very strong correlation between predicted and measured values, and thus confirmed the quality of the general predictions.

R_d_ measurements using an O_2_ electrode can be quick, around 2 min, allowing semi-HTP screening (O’Leary et al., 2017; Scafaro et al., 2017). However, the equipment is expensive and the technique requires destructive sampling of leaf material. To solve these problems, Coast et al. (2019) adapted a piecewise linear regression splines (PLRS) model based on equations developed by Serbin et al. (2012) to estimate R_d_ from large and diverse sets of wheat cultivars. In their experiment, several wheat cultivars were tested under controlled and field conditions thus analyzing a total of 1,318 leaf samples using a standard R_d_ measurement and hyperspectral reflectance measurements (Coast et al., 2019). These authors found an overall R^2^ between the measured and the predicted parameters of 0.50−0.63, which was higher than previous parameters used to estimate respiration such as leaf mass area (Wright et al., 2006) and leaf N content (Reich et al., 2008). As for V_cmax_ and J_max_, it was theorized that some of the low predictability of the models could be due to low phenotypic differences for R_d_. Indeed, low phenotypic variation has been identified as one of the problems when producing prediction models with NIRS technology as seen for the case of isotopic ratios (Cabrera-Bosquet et al., 2011). This limitation can be solved only by performing experiments with a diverse genetic background under different environmental conditions, or even better with stresses such as drought, elevated O_3_, increasing temperatures, etc. that will increase the variability of the measured phenotype. Furthermore, a collaborative database sharing phenotypes and spectroscopy data could advance this technology much quicker, as suggested by Coast et al. (2019).

Although further validation is needed using other species and under other environmental conditions such as drought and high temperature, this could be the beginning of an era where researchers can estimate gas exchange related parameters using hyperspectral reflectance spectroscopy data that only takes 1−2 min to collect. Until then, if a researcher is thinking of performing large cultivar screenings using values estimated from hyperspectral reflectance data, it is still recommended to have a reduced set of samples that serve to undertake gold standard measurements (A-Ci curves, or R_d_ measurements) just to test whether predictions are coherent. For example, a solution would be to measure and compare hyperspectral data with standard measurements using cultivars identified as extremes with hyperspectral data just to test that standard measurements identify them as extremes.

### High-Throughput Phenotyping Methods to Estimate Chlorophyll Fluorescence Parameters

Chlorophyll fluorescence measurements are based on capturing and measuring the light re-emitted by chlorophylls during a return from an excited to a non-excited state. Researchers measure chlorophyll fluorescence using different approaches: (1) After the leaf has been stimulated by solar radiation, and called “Solar Induced Fluorescence” (SIF). (2) After stimulation of the leaf with a light beam of known intensity and wavelength, and measurement at specific wavelengths, here referred to as “chlorophyll fluorescence.”

#### Solar Induced Fluorescence

As previously mentioned, reflected light from vegetation can provide information about various plant traits. Light reflected from plants contains light remitted by chlorophyll that contributes to the reflectance signature. Chlorophyll remits absorbed light (fluorescence) at peak wavelengths of 690 and 740 nm associated with PSII and PSI, respectively (Krause and Weis, 1984). The reflectance signature of leaves is an outcome of various parameters that influence how incoming radiation is reflected. The deconvolution of reflectance and fluorescence can be observed in absorption bands of oxygen (centered at 687 and 760.4 nm) and hydrogen (centered at 656.4 nm) where solar radiation is absorbed by the atmosphere (Meroni et al., 2009). Reflectance recorded near these wavebands is from chlorophyll fluorescence and thus, it is possible to passively measure the amount of fluorescence being emitted from plant tissues while solar radiation is reaching the plants.

Multispectral measuring methods of SIF require the incident solar irradiance to be obtained along with the vegetative reflectance after which SIF is calculated by comparing the relative increase between a wavelength in and out of the absorption band (Carter et al., 1990). SIF can be also calculated using hyperspectral reflectance spectroscopy as it contains more information in the multitude of wavelengths (Alonso et al., 2008). However, the fluorescence measured under these conditions is a complex outcome of physiological processes. Previous studies have shown how SIF can be used to obtain information about photosynthesis (Rosema et al., 1998; Flexas et al., 2002; Evain et al., 2004). SIF can be measured remotely by satellites and at nearer to ground levels using multispectral and hyperspectral platforms. These multispectral and hyperspectral methods are especially amenable to high-throughput analyses and can be incorporated into different HTP platforms such as aerial drones (Sankaran et al., 2015; Kanning et al., 2018), tractors (Scotford and Miller, 2004; Andrade-Sanchez et al., 2014), and carts (Thompson et al., 2018). Previously, SIF had been shown to be correlated with canopy photosynthesis (Yang et al., 2015) and used to estimate gross primary productivity (Bacour et al., 2019). Passive measurements of photosynthetic traits like SIF can be carried out extremely rapidly and at multiple times during the growing season.

#### Chlorophyll Fluorescence

Chlorophyll fluorescence is an important tool used to investigate the light-dependent reactions of photosynthesis. This is achieved by removing or drastically decreasing one of the three routes of absorbed light energy. Without the addition of herbicides that inhibit PSII, this is achieved by applying a short saturating flash to the photosynthetic sample. With a short enough flash, no changes to non-photochemical quenching or photosynthetic efficiency occur and this allows the fluorescence maximum to be reached that can, with other fluorescence measurements, provide information about PSII efficiency (Maxwell and Johnson, 2000).

The commercial availability of handheld fluorometers has allowed researchers to use chlorophyll fluorescence measurements to study the effects of various stresses on the light-dependent reactions including nitrogen availability (Huang et al., 2004), salinity (Belkhodja et al., 1994), heat (Pastenes and Horton, 1996), cold (Fracheboud et al., 1999), and drought (Meyer and Genty, 1999; Sánchez-Rodríguez et al., 1999). While the use of such fluorometers in the field has yielded valuable information, throughput is limited by the time required to walk from one plant to another and to initiate a new measurement. Additionally, the time frame in which photosynthetic traits are somewhat stable limits when measurements can be collected depending on the aim of the experiment. Because chlorophyll fluorescence is changing in response to irradiance, large data collections that span several hours can be influenced by when measurements were obtained (Huang et al., 2006). To avoid incorporating a large source of error, timing the measurements around solar noon, when chlorophyll fluorescence is relatively stable, produces better quality data. That said, several chlorophyll fluorescence studies involving large populations of genotypes have provided genetic information that could be used to improve photosynthesis and crop production (Guo et al., 2008; Kiani et al., 2008; Azam et al., 2015; Herritt et al., 2018).

Imaging-based methods for measuring chlorophyll fluorescence allow spatial details of leaf and plant canopy fluorescence that handheld devices cannot provide. This approach requires that the whole imaging area is provided with a rapid, homogenous, and saturating light flash. Thus, the field of view for the imaging system will dictate the number of light sources required to saturate the leaf area being imaged. Several studies have shown the sensitivity of fluorescence imaging concerning pathogen interactions (Meyer et al., 2001; Chaerle et al., 2004, 2007). More recently, several companies have offered systems that can obtain chlorophyll fluorescence images. However, the deployment of these and other custom-built systems in field experiments is often difficult. To achieve a high-throughput capacity with fluorescence imaging, automated systems that move the imaging system to the plants or move the plants to the imaging system are required (Fahlgren et al., 2015; Virlet et al., 2017). With the incorporation of such automated systems, chlorophyll fluorescence imaging can provide spatial information about the efficiency of the light-dependent reactions within large plant populations.

One emerging improvement in chlorophyll fluorometry is the use of light-emitting diodes (LEDs) to provide fast and repetitive flashes of sub-saturating light to obtain information about the primary electron acceptor of PSII as well as the reduction of the PQ pool. Previous fluorescence measurement methods relied on saturating light pulses to measure the relative changes in fluorescence required to describe biophysical and physiological aspects of photosynthesis (Avenson and Saathoff, 2018). The use of LEDs has allowed the development of multiphase flash techniques that use short sub-saturating light flashes to achieve a complete reduction of PSII primary quinone acceptors and PSII acceptor pools (Loriaux et al., 2013). Multiphase flash chlorophyll fluorescence allows for a more accurate measurement of light-adapted maximum fluorescence (F_m_’). Despite these improvements, the multiphase flash technique has not been incorporated into HTP. The potential for high-throughput measurements has been realized with the fast repetition rate (FRR) protocol thus allowing for extremely rapid measurements of fluorescence (<0.2 s) (Kolber et al., 1998). The combination of LED systems with FRR capability into laser or light-induced fluorescence transient (LIFT) instruments can provide high-throughput fluorescence data. Thus, LIFT systems have been incorporated into HTP systems and used in the field and controlled environments to collect plant fluorescence data (Keller et al., 2018).

## Modeling Photosynthesis in Crop Growth Models

Over the last five decades, many crop growth models have been developed and applied to simulate agricultural production systems and to forecast crop yields (Priesack and Gayler, 2009). In particular, during the last years, a selection of these models have been tested and compared to characterize their ability to simulate crop production at different sites across the globe situated in different continents and representing different climatic conditions for major crops such as wheat (Asseng et al., 2013), maize (Bassu et al., 2014), rice (Li T. et al., 2015), and potato (Fleisher et al., 2017). The aim was also to apply the models to estimate possible future impacts of a changing climate on global crop production and grain yields (Asseng et al., 2019; Liu Y. et al., 2019).

Almost all crop growth models aim to estimate the carbon gain for biomass production at the field level based on models of photosynthesis and radiation absorbed by the canopy. Many models assume a linear relationship between net primary biomass production (NPP) and the photoactive radiation absorbed by the crop canopy (*R*_PAR_). Models such as APSIM, CERES, EPIC, SALUS, LINTUL, Sirius, and STICS (Asseng et al., 2013 supplement, Bassu et al., 2014 supplement) follow the so-called “big-leaf” approach, where the whole canopy is treated as if it was one big leaf and photosynthetic carbon gain is described by the light-use-efficiency model. It is defined by the following direct proportionality with the parameterε_LUE_, the light-use-efficiency, representing all photosynthetic and respiratory processes (Medlyn, 1998):

(1) $$NPP=\varepsilon_{\text{LUE}}R_{\text{PAR}}$$

where NPP denotes net primary production [g m^–2^ d^–1^], *R*_PAR_ is absorbed photoactive radiation [MJ m^–2^ d^–1^] and ε_LUE_ is light-use-efficiency [g MJ^–1^].

Other models such as Ecosys, ExpertN-SPASS, GECROS, HERMES, IXIM, LPJml, MCWLA, MONICA, SUCROS, WOFOST (Asseng et al., 2013 supplement, Bassu et al., 2014 supplement) simulate the photosynthesis rate of the canopy based on single leaf photosynthesis rates. This is achieved in three major steps by calculating (i) single leaf photosynthesis per leaf area of each leaf, (ii) the instantaneous photosynthesis rate of the whole canopy at given light conditions by integration over the canopy depth and plant leaf areas at each depth, (iii) the daily canopy photosynthesis by integration over the day. In these models, a distinction is made between shaded and sunlit leaves (Spitters, 1986; Goudriaan and van Laar, 1994; Wang and Leuning, 1998) and leaf photosynthesis is calculated for each leaf type separately. This modeling approach is known as the “two-leaf” model.

A further type of canopy photosynthesis model distinguishes different leaf classes depending on their height in the canopy above the soil surface and it is called the “multi-layer” model (Leuning et al., 1995).

Besides the availability of light and CO_2_, the impact of air temperature and the supply of water and nitrogen on leaf photosynthesis have to be modeled depending on the modeling approach at either canopy or both at the leaf and canopy-scales.

### Leaf Photosynthesis Rate Models

In the case of the “big-leaf” approach, as with the LINTUL model, the daily net gain of carbon for biomass growth is described by:

(2) $$\mu_{B}=\varepsilon_{LUE}\cdot \left\{R_{PAR}\left[1-\exp\left(-\alpha_{ext}\cdot f_{LAI}\right)\right]\right\}\cdot f_{CO_{2}}\cdot f_{S}\cdot f_{T}\cdot f_{\vartheta }\cdot f_{N}$$

where μ_B_ is the daily net carbon gain of the canopy biomass [g m^–2^ d^–1^], ε_LUE_ the light-use-efficiency [g MJ^–1^], *R*_PAR_ the absorbed photoactive radiation [MJ m^–2^ d^–1^], α_ext_ the light extinction coefficient, *f*_LAI_ the leaf area index, *f*_CO_2__ the impact factor of atmospheric CO_2_ concentration, *f*_S_ the impact factor of senescence, *f*_T_ the impact factor of daily average air temperature, *f*_ϑ_ the impact factor of available soil water content, and *f*_N_ the impact factor of available soil nitrogen. In a similar way this approach is used in the CERES model, where only the term representing the absorbed global radiation takes an empirically derived exponential form, i.e:

(3) $$\begin{array}{c} \mu_{B}=\varepsilon_{\text{LUE}}\cdot {\left\{R_{\text{PAR}}\left[1-\exp\left(-\alpha_{ext}\cdot f_{\text{LAI}}\right)\right]\right\}}^{0.6}\\ \;\cdot f_{{\mathrm{CO}}_{2}}\cdot f_{S}\cdot f_{T}\cdot f_{\vartheta }\cdot f_{\text{N}} \end{array}$$

In cases of the “two-leaf” or the “multi-layer” approach, the description of leaf photosynthesis rates again follows the general scheme given by a maximal rate of carbon gain and additional reduction factors representing environmental conditions which are not often in an optimal state to allow maximal photosynthesis:

(4) $$P_{gm}=P_{g\max}\cdot f_{{\mathrm{CO}}_{2}}\cdot f_{S}\cdot f_{T}\cdot f_{\vartheta }\cdot f_{\text{N}}$$

where *P*_gm_ denotes the gross leaf photosynthesis rate at light saturation [kg CO_2_ m^–2^ d^–1^] and *P*_gmax_the maximal gross leaf photosynthesis rate [kg CO_2_ m^–2^ d^–1^] with impact factors of CO_2_, senescence S, temperature T, soil water ϑ and soil nitrogen availability *N*.

This scheme is similar to the mechanistic description of growth rates, which change by the impact of environmental factors, formulated in analogy to mechanics, i.e., to the description of the velocity change of a moving particle due to forces acting on the particle (Priesack et al., 2012).

The gross leaf photosynthesis rate *P*_gl_[kg CO_2_ m**^–^**^2^ d**^–^**^1^] is then given by accounting for the absorbed photoactive radiation *R*_PAR_ [MJ m**^–^**^2^ d**^–^**^1^] and by applying the light-use-efficiency parameter ε_PAR_[kg CO_2_ MJ**^–^**^1^] of photosynthesis and the gross photosynthesis rate at light saturation *P*_gm_[kg CO_2_ m**^–^**^2^ d**^–^**^1^]:

(5) $$P_{gl}=P_{gm}\left\{1-\exp\left(-\varepsilon_{\text{PAR}}\cdot R_{\text{PAR}}P_{gm}^{-1}\right)\right\}$$

### Whole Canopy Photosynthesis Rate Models

The up-scaling calculation from leaf photosynthesis rate to whole canopy photosynthesis rate for a given time during the day often follows the method of Spitters et al. (1989). It is assumed that light-use-efficiency of photosynthesis ε_PAR_ and gross photosynthesis at light saturation *P*_gm_ are constant within the canopy. In a first step, photosynthesis rates of shaded and sunlit leaves at each depth of the canopy are calculated separately. In the case of sunlit leaves, an additional integration over the leaf angle distribution is performed to include an averaged value of the adsorbed direct radiation for the estimation of sunlit leaf photosynthesis rates at different canopy depths. Finally, the integration over the canopy depth of the photosynthesis rates of both sunlit plus shaded leaves gives gross canopy photosynthesis at any given time during the day.

*P*_g,l,z_ [g CO_2_ m**^–^**^2^ d**^–^**^1^] denotes the total photosynthesis at depth z of the canopy given by the fraction of sunlit leaves *f*_slt,z_ at depth z and the gross photosynthesis of sunlit *Pg*, *slt*, *z* or shaded leaves *P*_g,shd,z_:

(6) $$P_{g,l,z}=f_{slt,z}P_{g,slt,z}+\left(1-f_{slt,z}\right)P_{g,shd,z}$$

Since the integration over the cumulative leaf area index *f*_LAI,z_ at canopy depth z from zero at the soil surface to the total leaf area index *f*_LAI_ of the canopy corresponds to the integration over the canopy height, the total gross photosynthesis of the canopy *P*_*g*_ can be calculated using:

(7) $$P_{g}=\int_{0}^{f_{\text{LAI}}}P_{g,l,z}df_{\mathrm{LAI},z}$$

The daily gross photosynthesis and hence the daily amount of assimilated carbon by the canopy is then estimated by integration over the day length (Spitters et al., 1989) from the time of sunrise *t*_0_ to the time of sunset *t*_1_:

(8) $$P_{g}^{day}=\int_{t_{0}}^{t_{1}}P_{g}dt$$

Gaussian integration is usually applied as a fast and accurate method to calculate both instantaneous and daily canopy photosynthesis (Goudriaan, 1986; Spitters, 1986).

In the case of the GECROS model, upscaling from the leaf transpiration as determined by leaf stomatal conductance from either sunlit or shaded leaves to the whole canopy transpiration is achieved by the same integration procedures (Yin and van Laar, 2005).

### Impact Factors of Temperature, Atmospheric CO_2_ Concentration, Soil Water, and Soil Nitrogen Availability

Besides the differences between “big-leaf,” “two-leaf,” and “multi-layer” approaches, crop growth models mainly differ in their choice of impact functions.

The impact functions of air temperature are well documented in the supplementary information of Wang et al. (2017) an will not be mentioned further here.

To simulate the impact of atmospheric CO_2_ concentration on photosynthesis, strongly different approaches have been incorporated into crop growth models especially if CO_2_ enrichment experiments are considered. In the case of the “big-leaf” approach, the CO_2_ impact factor is either a linear or a curvilinear multiplier leading to an increase of light-use-efficiency (Tubiello and Ewert, 2002), as in several models including CERES, Cropsyst, EPIC, Sirius and STICS (Vanuytrecht and Thorburn, 2017). In the case of leaf scale photosynthesis models, common and often documented approaches are the biochemical model of leaf photosynthesis of Farquhar et al. (1980) for C_3_ plants and an equivalent version by Yin and Struik (2009) for C_4_ plants. Both models are based on the calculation of intercellular CO_2_ concentration and require the incorporation of a stomatal conductance model. However, for both photosynthesis models, several parameters have to be determined and the application of the model can be difficult. A simpler approach for leaf-level photosynthesis is the empirically determined increase of light-saturated photosynthetic rate prescribed by the impact factor *f*_CO_2_ in eq. (4). Additionally, photosynthetic light-use-efficiency ε_PAR_ can be modeled as influenced by atmospheric CO_2_ concentrations (Nendel et al., 2009) and it is increased if higher CO_2_ concentrations occur (Vanuytrecht and Thorburn, 2017). In contrast, the more complex structure of the GECROS model can simulate the acclimation of photosynthesis to higher CO_2_ levels, which has been observed in FACE experiments. This good model performance is based on a better consideration of plant internal C−N interactions (Thornley, 1998, 2004) within the GECROS model (Biernath et al., 2013; Yin, 2013).

In several models, the impact factor of water availability on photosynthesis is set equal to the ratio of actual to potential evapotranspiration, which then reduces canopy light-use-efficiency or the maximal leaf photosynthesis rate if the actual transpiration, i.e., the root water uptake, is less than the potential demand. It is assumed that stomatal closure is controlled by the balance between available soil water and water demand caused by atmospheric conditions (Tubiello and Ewert, 2002). Less simple approaches calculate leaf stomatal conductance, which decreases under water stress, and thus limits photosynthetic rates by reducing CO_2_ entry into leaves or fluxes within the canopy. This has to be based on simulations of energy balance either at the leaf or canopy level to adequately represent the impact of atmospheric conditions that determine transpiration demand.

Similar to the very simple approach to account for water availability, the impact factor of nitrogen availability can be defined as the ratio of actual nitrogen demand versus optimal nitrogen supply. This is often calculated as the ratio of the difference between actual and minimal nitrogen content about the difference between optimal and minimal nitrogen content of either the leaf in the case of leaf photosynthesis or of the aboveground canopy biomass in the case of canopy photosynthesis. It is assumed that nitrogen contents are not optimal if the mineral nitrogen uptake from the soil cannot fulfill plant nitrogen demand given as the sum of the differences between actual and optimal concentrations in the plant organs (Priesack and Gayler, 2009). More complex nitrogen uptake models can also simulate the observed increase in photosynthetic nitrogen-use-efficiency and decreased tissue N concentrations at elevated [CO_2_] (Biernath et al., 2013; Vanuytrecht and Thorburn, 2017). This is achieved for example by incorporating a functional balance between root N acquisition and shoot C gain in GECROS (Yin and van Laar, 2005; Priesack and Gayler, 2009), or by including an adaptation of photosynthetic N demand to atmospheric [CO_2_] as in the growth model AgPasture of APSIM (Cullen et al., 2009).

Most of the considered crop models are source driven assuming growth limitation by the supply of assimilates. Therefore, approaches to model either positive or negative environmental impacts on yields by factors increasing or reducing maximal leaf photosynthesis rate or crop light-use-efficiency strongly determine the simulation of crop growth. Determination of these factors needs numerous field experiments and extensive testing to provide a sound basis for adequate simulations of impacts on crop growth. By this rather simple and parsimonious approach, crop growth is conceived as carbon-source driven and described by balancing gains from assimilation and losses through respiration and plant tissue abscission.

## Metabolic Engineering to Improve Photosynthesis and Elevated CO_2_ Acclimation

**FIGURE 4:**
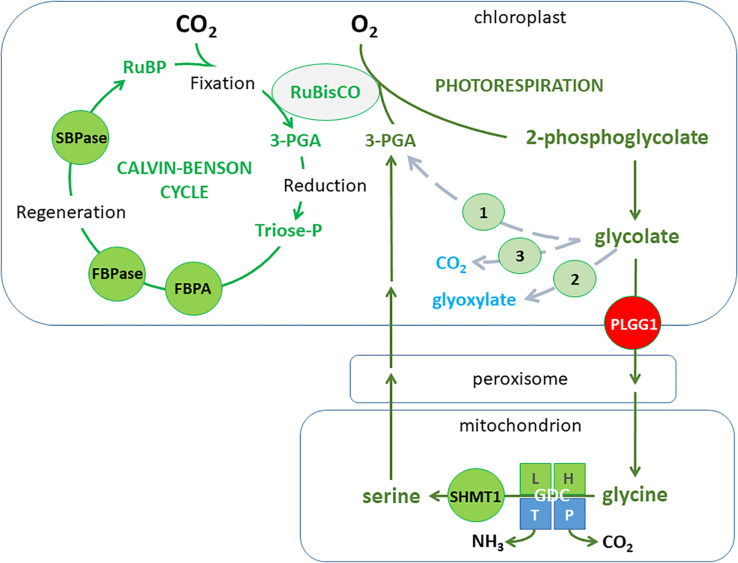
A simplified scheme highlighting tested ways to increase photosynthesis and crop yield by overexpressing selected Calvin cycle enzymes and by creating synthetic chloroplastic photorespiratory bypasses. The enzymes in green circles/squares have been overexpressing either individually or together. Details of the different photorespiratory pathways can be found in the main text: 1 is based on the bacterial glycerate pathway leading to 3-PGA from glycolate (Kebeish et al., 2007; Dalal et al., 2015), 2 is an incomplete glycerate pathway leading to glyoxylate from glycolate (Nölke et al., 2014), 3 are pathways leading to the production of CO_2_ from glycolate (Maier et al., 2012; Shen et al., 2019; South et al., 2019). The transporter in the red circle was repressed to improve photorespiratory bypasses (see South et al., 2019). Abbreviations can be found in the main text.

As already stated in this review, improving photosynthesis has become a major aim for increasing plant yield (example: the RIPE project^2^) (Long et al., 2015; Ort et al., 2015; Simkin et al., 2019; Weber and Bar-Even, 2019). To date, targets to achieve this include: RuBisCO properties and activation, RuBP regeneration, photorespiration, CO_2_ availability by improving mesophyll conductance and by introducing CO_2_ concentrating mechanisms based on cyanobacterial, algal and C4-plant systems, photoprotection by modifying the relaxation of energy quenching processes, and by optimizing crop canopies to improve light capture (see Ort et al., 2015). Already, several studies have provided support by demonstrating that modifying photosynthetic processes through genetic engineering can improve photosynthetic CO_2_ assimilation rates and yield potential (see reviews by Simkin et al., 2019; Weber and Bar-Even, 2019). Several major examples are highlighted below and include improving RuBP regeneration by overexpressing selected Calvin cycle enzymes and modifying photorespiration by creating artificial glycolate-metabolizing bypass pathways in the chloroplast (see Figure 4). These processes were found to be amongst the best targets to improve photosynthesis CO_2_ assimilation efficiency after *in silico* modeling studies pin-pointed SBPase, fructose bisphosphate aldolase (FBPA), and photorespiration as potential limiting reactions (Zhu et al., 2007).

Plants over-expressing the redox-regulated Calvin cycle enzyme SBPase show improved photosynthetic activities and increased biomass. This has been seen to occur in *Arabidopsis thaliana* (Simkin et al., 2017a), tobacco (Lefebvre et al., 2005; Rosenthal et al., 2011; Simkin et al., 2015), tomato (Ding et al., 2016), and wheat (Driever et al., 2017). However, beneficial effects were found to be dependent on both developmental stage and growth conditions. An increase in photosynthesis was only observed in young expanding tobacco leaves but not in fully expanded ones and no effect on photosynthesis was seen when plants were grown under short days and low light (Lefebvre et al., 2005). When tobacco over-expressing Arabidopsis SBPase was grown outside under elevated CO_2_ (585 ppm) an increase in photosynthesis and biomass was observed when compared to wild-type plants (Rosenthal et al., 2011). However, the increase in biomass was only 50% of the theoretical value due to C3-plant acclimation to elevated CO_2_ (see below). Furthermore, higher CO_2_ assimilation rates were variable over the growing season with no significant increase observed in August compared to July. Cyanobacterial and green algal Calvin cycle enzymes have also been used to improve plant productivity. The overexpression of either Chlamydomonas SBPase or cyanobacterial FBPase led to increases in both photosynthesis and biomass (Tamoi et al., 2006). A cyanobacterial bifunctional SBPase/FBPase enzyme has been overexpressed also in tobacco (Miyagawa et al., 2001), lettuce (Ichikawa et al., 2010), and soybean (Köhler et al., 2017) with increases in photosynthetic CO_2_ fixation and biomass. In the work of Köhler et al. (2017), soybean overexpressing cyanobacterial SBPase/FBPase was grown in the field during three seasons under elevated CO_2_ (600 ppm) and elevated temperature (+3°C) and compared to normal ambient conditions. Across the different treatments, the over-expressing lines exhibited higher carbon assimilation rates. Under ambient CO_2_, elevated temperature led to seed yield reductions in both control and transgenic genotypes. However, under elevated CO_2_ and high temperature, the SBPase/FBPase plants maintained higher seed yield levels, while WT plants had reduced seed yields, compared with plants grown under only high CO_2_. Therefore, perhaps Calvin cycle manipulation can offset the detrimental effects of future climate change conditions. Improved biomass has been observed also when overexpressing FBPA in tobacco (Uematsu et al., 2012) and the positive effect on photosynthesis and biomass was more pronounced when plants were grown at elevated CO_2_ (700 ppm). When Arabidopsis FBPA was overexpressed in the photosynthetic tissues of Arabidopsis using a RuBisCO small subunit 2A promoter, similar increases in photosynthesis, dry weight, and seed yield occurred (Simkin et al., 2017a). However, when overexpression of SBPase and FBPA were stacked in Arabidopsis, no significant additional increases in maximal efficiency of CO_2_ assimilation rate (A_max_), dry weight and seed yield were observed when compared to individual transgene overexpressing lines (Simkin et al., 2017a).

Another major strategy for improving photosynthesis has been a synthetic biology approach to express within the chloroplast an alternative pathway to efficiently metabolize photorespiratory glycolate and thus alleviate the potential inhibitory action of 2-PG on photosynthesis, while preventing ammonium release and limiting CO_2_ release to the chloroplast where RuBisCO is located (Figure 4). Since the first report (Kebeish et al., 2007), several photorespiratory bypass strategies have been used to improve photosynthetic CO_2_ assimilation and biomass. The initial bypass tested was based on the bacterial glycerate pathway where glyoxylate was converted to glycerate by two enzymes; glyoxylate carboligase and tartronate semialdehyde reductase (Figure 4, pathway 1). The conversion of glycolate to glyoxylate in the chloroplast was achieved by expressing a bacterial glycolate dehydrogenase (GlycDH). When expressed in Arabidopsis chloroplasts, transgenic lines produced bigger rosettes and more biomass while increasing A_max_ (Kebeish et al., 2007). A similar strategy was used to increase biomass and seed yield in *Camelina sativa* (Dalal et al., 2015). Bacterial GlycDH alone was introduced into *Solanum tuberosum* using a single construct to produce a polyprotein to circumvent instability problems (Nölke et al., 2014). These potato lines exhibited an enhanced A_max_ at 400 ppm CO_2_ (but not when measured at 2000 ppm CO_2_) and an increase in tuber yield even though the complete glycerate pathway had not been introduced (Figure 4, pathway 2). This was observed also for GlycDH-expressing Arabidopsis (Kebeish et al., 2007) and *Camelina sativa* (Dalal et al., 2015). Another successful photorespiratory bypass was achieved by expressing glycolate oxidase, catalase and malate synthase in chloroplasts (Maier et al., 2012). Such a bypass has the potential to completely oxidize glycolate to CO_2_ and it led to increases in leaf dry weight and net CO_2_ fixation rates (Figure 4 pathway 3). More recently, another chloroplastic photorespiratory bypass (named the GOC bypass) was expressed in rice to increase photosynthetic efficiency (Shen et al., 2019). It consisted of three rice enzymes; glycolate oxidase, oxalate oxidase, and catalase expressed in chloroplasts and designed to lead to the complete oxidation of glycolate to CO_2_ (Figure 4 pathway 3). Improved photosynthetic efficiency, biomass, and yield were found in both greenhouse and field experiments although there were differences according to seeding season and it was more advantageous under high light. To improve flux through chloroplastic photorespiratory bypasses, glycolate export out of the chloroplast was manipulated by down-regulating PLGG1 (a plastidial glycolate/glycerate transporter, Pick et al., 2013; South et al., 2019). The best results were obtained with a variant of the glycolate oxidation pathway where glycolate oxidase was replaced by *Chlamydomonas reinhardtii* GlycDH (Figure 4, pathway 3). Field-grown tobacco expressing this version of an alternative photorespiratory pathway exhibited a >25% increase in total vegetative biomass (without PLGG1 inhibition) and a 40% increase (with inhibited PLGG1) although the impact on net CO_2_ assimilation was quite low (5−8%) and no significant increase in seed yield was observed (South et al., 2019).

The over-expression of specific photorespiratory enzymes has also led to increased biomass, A_max_, and grain yield. This was observed when mitochondrial serine hydroxymethyltransferase (SHMT1) was overexpressed in rice (Wu et al., 2015) and when individual glycine decarboxylase (GDC) subunits were overexpressed in Arabidopsis either the H-protein (Timm et al., 2012) or the L-protein (Timm et al., 2015; Figure 4). When the H-protein was overexpressed in tobacco, improved biomass was only observed when under the control of a leaf-specific promoter and this only became significant at high light intensities while constitutively overexpressed H-protein led to a detrimental growth effect (López-Calcagno et al., 2019). In gene stacking experiments, the additional overexpression of the H-protein in Arabidopsis lines overexpressing SBPase and FBPA led to further improvements in seed weight per plant (under high light growth conditions) and leaf area with no further increase in A_max_ compared to SBPase-FBPA lines alone (Simkin et al., 2017a).

Improved photosynthesis has also been found in plants where the light-side of photosynthesis has been manipulated. In Arabidopsis, the overexpression of the Rieske-FeS protein of the cytb_6_f complex led to plants exhibiting increased A_max_, dry weight, leaf area and seed yield (Simkin et al., 2017b). Photosynthesis was improved also under fluctuating light conditions by overexpressing violaxanthin de-epoxidase, zeaxanthin epoxidase and *PsbS*, all components of a photoprotection mechanism involving light energy dissipation as heat (Kromdijk et al., 2016). Field-grown tobacco plants overexpressing these three proteins showed increases in dry weight, leaf area and plant height (Kromdijk et al., 2016).

As mentioned above, certain strategies to improve photosynthesis and yield have already been tested under one or more climate change condition(s) such as elevated temperature and CO_2_ levels. In general, increased temperatures of only several °C have been shown to cancel the beneficial effects of elevated CO_2_ (see Köhler et al., 2017). Multiple FACE experiments (carried out at around 600 ppm CO_2_) have consistently shown that the increase in C3-crop yield in response to long-term elevated CO_2_ conditions is 50% lower than predicted due to photosynthetic acclimation (see Ainsworth and Long, 2005; Kant et al., 2012). Many C3-plant species only exhibit a small 15% increase in yield compared to a hypothetical 40% increase under predicted climate change CO_2_ levels. This yield response to elevated CO_2_ has been observed in both controlled growth conditions and FACE experiments (see Leakey et al., 2009 and references therein) where a significant reduction in N-content was also reported (Ainsworth and Long, 2005; Vicente et al., 2015). This underachievement of certain C3 model plants like *Arabidopsis thaliana* and major C3-cereal plants including wheat and rice to elevated CO_2_ is due to modifications in plant metabolism, physiology, and development where acclimation is associated with a negative impact on leaf photosynthesis (Ainsworth and Rogers, 2007), root nitrate uptake and leaf nitrate assimilation (Rachmilevitch et al., 2004; Bloom et al., 2010), thus reducing the expected benefits of elevated CO_2_. It includes a reduction in RuBisCO protein content, a reduction in stomatal conductance, and decreases in both photosynthetic and N-assimilation gene expression (e.g., Vicente et al., 2015) that brings about a reduction in leaf and seed N-content. In the literature, photosynthetic acclimation to elevated CO_2_ has been explained by the inhibition of photosynthetic gene expression due to the accumulation of excess non-structural carbohydrates in source leaves (Moore et al., 1999; Ruiz-Vera et al., 2017). However, it is possible that this acclimation is also driven by N-limitations when N-assimilation cannot keep up with the increased C-assimilation rates. It has also been suggested that at elevated CO_2_, a decrease in photorespiration impacts negatively both nitrate uptake and assimilation (Bloom, 2015). Several factors have been proposed to influence acclimation to elevated CO_2_, such as sink strength, sugar signaling, and N-regime (see Long et al., 2004 and references therein). Several papers have suggested a link between improved sink strength and a reduction of this acclimation in tobacco (Ruiz-Vera et al., 2017), rice (Zhu et al., 2014), barley (Torralbo et al., 2019), and *Larrea tridentata* (Aranjuelo et al., 2011). Although a number of actors in sugar signaling and sensing are known (see Pego et al., 2000), a lack of information on how they are affected by elevated CO_2_, especially in roots, has been stated (Thompson et al., 2017). This is similar to nitrate-signaling where actors of perception, signal transduction and even root to shoot communication have been discovered (see Wang et al., 2018) but little is known about how elevated CO_2_ and other climate change factors affect such processes. Indeed, to date, there is no global understanding of the regulatory networks involved in the acclimation processes occurring to balance plant C and N metabolism under elevated CO_2_. That said, a recent work using correlation network analyses confirmed the tight coordination between C and N metabolisms while carbohydrate levels were linked to the down-regulation of both photosynthetic and N metabolism genes (Vicente et al., 2018).

When light is saturating, photosynthesis can be limited by several factors including V_cmax_ (amount and maximal carboxylase activity of RuBisCO), RuBP regeneration, triose-phosphate utilization/carbohydrate export (source-sink properties) and, of course, photorespiration. Under future climate conditions of elevated CO_2_ and temperature, major limitations will probably shift to RuBP regeneration and source-sink properties. Under elevated CO_2_, plants reduce RuBisCO amounts since it is no longer a limiting factor but they need to improve their photosynthetic electron transport properties to produce enough NADPH and ATP to regenerate RuBP via the Calvin cycle. Less RuBisCO is a common feature of elevated CO_2_ acclimation in C3-cereals while N reallocations to improve the light reactions are not adequate and there is an overall reduction in plant N-content.

Predicted climate change conditions of elevated CO_2_ and temperature have been shown to affect the benefits of improved photosynthesis by current genetic manipulations, as mentioned above (Rosenthal et al., 2011; Köhler et al., 2017). That said, strategies used to improve RuBP regeneration have often given rise to the best increases in photosynthesis and yields under either elevated CO_2_ or high light or both (see above). However, strategies to reduce the negative impact of photorespiratory carbon recycling might be expected to have a lesser impact under conditions that lower photorespiration like elevated CO_2_ although benefits may still occur under elevated temperatures and high light in association with CO_2_. It is probable that C3-plant acclimation to future atmospheric CO_2_ and temperature levels could hamper strategies to improve photosynthesis and yield of actual plant genotypes. Therefore, perhaps additional strategies to reduce C3-plant acclimation by deregulating plant functions associated with known acclimation processes might be required. This would require extensive omics analyses to identify the regulating gene networks and proteins involved in photosynthetic acclimation to climate change conditions, this could be helped by photosynthetic performance measurements in the field using non-destructive HTP techniques and platforms while the data sets could be used to improve plant growth models to predict the benefits. In this way, the best gene targets will be identified and tested to create new crops for the future.

## Author Contributions

MB and ÁS-S integrated the contributions. All authors read and approved the final version of the manuscript.

## Conflict of Interest

The authors declare that the research was conducted in the absence of any commercial or financial relationships that could be construed as a potential conflict of interest.

## Footnotes

1 https://sites.google.com/site/ijmackay/work/magic

2 http://ripe.illinois.edu/
